# SARS-CoV-2 escapes direct NK cell killing through Nsp1-mediated downregulation of ligands for NKG2D

**DOI:** 10.1016/j.celrep.2022.111892

**Published:** 2022-12-12

**Authors:** Madeline J. Lee, Michelle W. Leong, Arjun Rustagi, Aimee Beck, Leiping Zeng, Susan Holmes, Lei S. Qi, Catherine A. Blish

**Affiliations:** 1 Stanford Immunology Program, Stanford University School of Medicine, Stanford, CA 94305, USA; 2 Department of Medicine, Stanford University School of Medicine, Stanford, CA 94305, USA; 3 Department of Bioengineering, Stanford University, Stanford, CA 94305, USA; 4 Department of Statistics, Stanford University, Stanford, CA 94305, USA; 5 Sarafan Chem-H, Stanford University, Stanford, CA 94305, USA; 6 Chan Zuckerberg Biohub, San Francisco, CA 94157, USA; 7 Stanford Medical Scientist Training Program, Stanford University School of Medicine, Stanford, CA 94305, USA

**Keywords:** COVID-19, SARS-CoV-2, natural killer cells, NKG2D, Nsp1, immune escape, antiviral immunity, innate immunity, NK cells

## Abstract

Natural killer (NK) cells are cytotoxic effector cells that target and lyse virally infected cells; many viruses therefore encode mechanisms to escape such NK cell killing. Here, we interrogate the ability of SARS-CoV-2 to modulate NK cell recognition and lysis of infected cells. We find that NK cells exhibit poor cytotoxic responses against SARS-CoV-2-infected targets, preferentially killing uninfected bystander cells. We demonstrate that this escape is driven by downregulation of ligands for the activating receptor NKG2D (NKG2D-L). Indeed, early in viral infection, prior to NKG2D-L downregulation, NK cells are able to target and kill infected cells; however, this ability is lost as viral proteins are expressed. Finally, we find that SARS-CoV-2 non-structural protein 1 (Nsp1) mediates downregulation of NKG2D-L and that Nsp1 alone is sufficient to confer resistance to NK cell killing. Collectively, our work demonstrates that SARS-CoV-2 evades direct NK cell cytotoxicity and describes a mechanism by which this occurs.

## Highlights

• SARS-CoV-2-infected cells are inefficiently killed by activated, healthy NK cells
• SARS-CoV-2 strongly downregulates the ligands for the activating receptor NKG2D
• SARS-CoV-2 protein Nsp1 mediates downregulation of NKG2D ligands
• Nsp1 alone is sufficient to confer significant resistance to NK-mediated killing

## Graphical abstract

**Figure:**
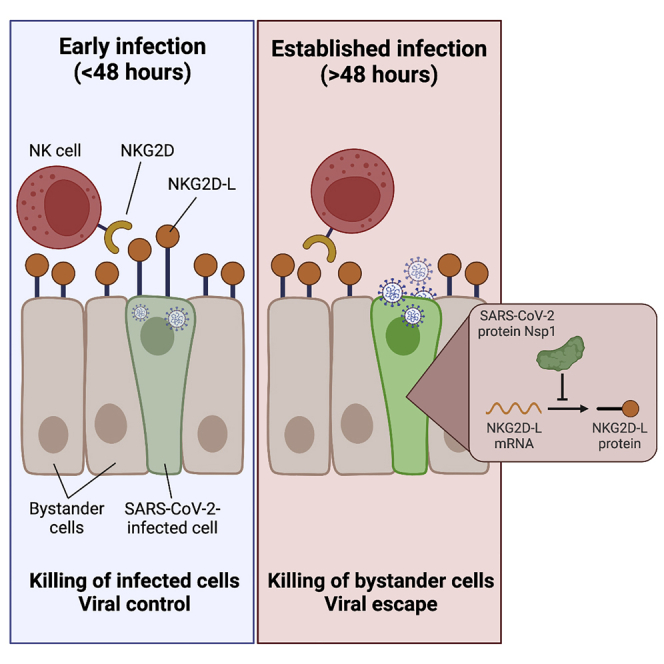

## Teaser

Natural killer (NK) cells are designed to kill virally infected cells. Lee et al. demonstrate that healthy NK cells are unable to efficiently lyse SARS-CoV-2-infected cells, likely due to their loss of the ligands for the activating receptor NKG2D. These effects are mediated by the viral protein Nsp1.

## Introduction

Natural killer (NK) cells are innate lymphocytes that play a critical role in the immune response to viral infection.^1^^,^^2^^,^^3^^,^^4^ Since the advent of the COVID-19 pandemic, studies examining the immune response in COVID-19 have noted that NK cells are less abundant in the peripheral blood of severe COVID-19 patients than in healthy donors^5^^,^^6^^,^^7^^,^^8^^,^^9^^,^^10^^,^^11^^,^^12^^,^^13^; a concurrent increase in NK cell frequency in the lungs of critically ill patients suggests that peripheral depletion of NK cells may be due to trafficking to the site of infection.^14^ In addition, immune profiling has uncovered significant, severity-associated phenotypic and transcriptional changes in the peripheral NK cells that remain in the blood of COVID-19 patients. In severe COVID-19, peripheral blood NK cells become activated and exhausted.^6^^,^^7^^,^^9^^,^^11^^,^^13^^,^^15^^,^^16^^,^^17^ They also downregulate surface level expression of the activating receptors NKG2D and DNAM-1, possibly as a consequence of internalization after ligation^7^^,^^10^ and exhibit defects in their ability to respond to tumor target cells and cytokine stimulation compared with NK cells from healthy donors.^11^^,^^13^^,^^15^

Less is known about how NK cells respond directly to SARS-CoV-2-infected cells, although several studies have demonstrated that NK cells can suppress SARS-CoV-2 replication *in vitro*.^16^^,^^18^^,^^19^ Moreover, a recent study found that NK cells are able to mount robust antibody-mediated responses against SARS-CoV-2-infected target cells.^20^ However, the mechanisms underlying NK cell responses to SARS-CoV-2-infected cells are not understood. This is particularly important because many viruses employ mechanisms that allow them to evade recognition and killing by NK cells. For example, both HIV-1 and human cytomegalovirus downregulate the ligands for NK cell activating receptors, shielding infected cells from recognition by NK cells.^21^^,^^22^^,^^23^^,^^24^^,^^25^^,^^26^^,^^27^^,^^28^^,^^29^^,^^30^^,^^31^^,^^32^

In this study, we utilized primary NK cells from healthy donors in conjunction with replication-competent SARS-CoV-2 to create an *in vitro* model system that dissects the NK cell response to SARS-CoV-2-infected cells. We focused on assessing the direct killing of infected target cells to better understand how the balance between SARS-CoV-2 recognition and escape contributes to disease. Our results demonstrate that SARS-CoV-2-infected cells efficiently escape killing by healthy NK cells, likely due to downregulation of ligands for the activating receptor NKG2D. Furthermore, we interrogated the mechanisms underlying this phenomenon and identified a specific SARS-CoV-2 protein, non-structural protein 1 (Nsp1), that mediates escape from NK cell recognition. Collectively, our work deeply interrogates the NK cell response to SARS-CoV-2 and provides insight into the role of NK cells in COVID-19.

## Results

### SARS-CoV-2-infected cells evade NK cell killing through a cell-intrinsic mechanism

**Figure 1:**
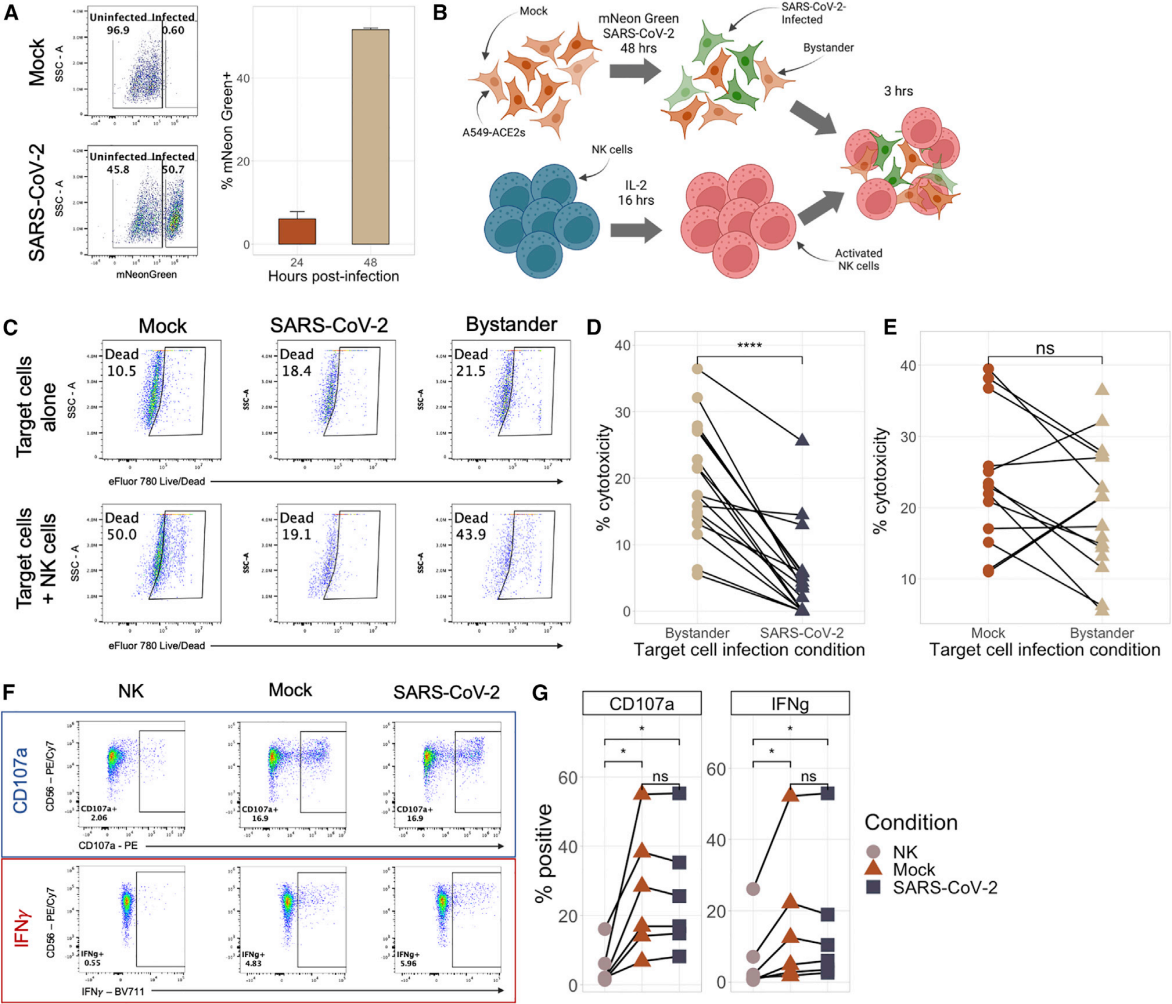
SARS-CoV-2-infected target cells evade NK cell killing through a cell-intrinsic mechanism (A) Representative flow plots (left) and boxplot (right) showing the percentage of mNeonGreen+ A549-ACE2 cells following infection with either mNeonGreen SARS-CoV-2 (MOI = 0.5) or media (mock) at an MOI of 0.5 for either 24 or 48 h. Bar plots represent mean of n = 4 technical replicates ± SD values. (B) Schematic illustrating the experimental design of NK cell killing assays. (C) Representative flow plots showing expression of eFluor 780 viability dye in target cells with NK cells (top) and with NK cells (bottom). (D and E) Background-subtracted percentage of A549-ACE2 cell death as measured by eFluor 780 viability dye staining in either infected versus exposed, uninfected cells (D) or mock-infected versus exposed, uninfected cells (E). Background cell death for each experiment and condition was calculated as the average level of death in four wells of the condition of interest. Data are shown from n = 18 unique healthy donors across 4 separate experiments. Lines connect points from individual donors. (F and G) Representative flow plots (F) and quantitations (G) of percentage of NK cells expressing CD107a and IFN-γ upon culture with no targets, mock-infected targets, or SARS-CoV-2-infected targets. Lines connect points from individual donors (n = 6). Significance values for all plots in this figure were determined using a paired Wilcoxon signed-rank test with the Bonferroni correction for multiple hypothesis testing.

We established a system to explore the NK cell response to SARS-CoV-2 infection using A549-ACE2 cells,^33^ which are lysed by NK cells and are infectible with SARS-CoV-2. We infected A549-ACE2 cells with SARS-CoV-2/WA1-mNeonGreen^34^ (which replaces ORF7a with mNeonGreen) at a multiplicity of infection (MOI) of 0.5 (Figure 1A). After 24 h, approximately 6% of cells fluoresced green, increasing to 50% by 48 h (Figure 1A). This suggests that, although SARS-CoV-2 only requires ∼8 h to complete its life cycle,^35^^,^^36^ 48 h is required for detection of robust viral protein expression in a low MOI system in which viral replication results in spreading infection. To understand how exposure to SARS-CoV-2-infected target cells impacts NK cell phenotype and function, we added NK cells from healthy donors that had been preactivated overnight with IL-2 to target cells that had been infected for 48 h (Figure 1B). This is an important distinction from previous studies that added NK cells early after SARS-CoV-2 infection, before the virus-infected cell expresses the full complement of viral proteins.^16^^,^^18^^,^^19^ We then assessed the ability of NK cells to directly lyse SARS-CoV-2-infected (mNeonGreen+) target cells compared with bystander (exposed but mNeonGreen−) and mock-infected cells (Figures 1C–1E).

NK cell co-culture induced significantly more death of uninfected “bystander” cells than of SARS-CoV-2-infected cells in all 18 NK cell donors tested (Figure 1C). We found no significant difference in the killing of bystander cells compared with mock-infected cells that were never exposed to SARS-CoV-2, indicating that the ability of SARS-CoV-2-infected cells to survive is a cell-intrinsic effect (Figure 1D). To ensure that these differences were not a result of rapid cell death resulting in cell loss and undercounting of killed SARS-CoV-2-infected cells, we assessed the ratio of infected (mNeonGreen+) target cells to uninfected (mNeonGreen−) target cells in cultures without NK cells compared with cultures with NK cells, gating only on “live” versus “total” cells. There was no difference in this ratio among all single cells (not live gated) in the presence and absence of NK cells, suggesting that if cells are disappearing from culture due to apoptosis, they are disappearing at an equal rate among infected and bystander cells (Figure S1). Meanwhile, the ratio of mNeonGreen+ cells to mNeonGreen− cells was increased in live-gated cells upon addition of NK cells due to preferential killing of uninfected target cells by NK cells (Figure S1).

### SARS-CoV-2-infected cells do not actively inhibit NK cell functionality

We next interrogated changes in NK cell phenotype and function induced by co-culture with mock- or SARS-CoV-2-infected target cells. Importantly, we continued utilizing an MOI of 0.5, resulting in around 50% infection of the SARS-CoV-2-infected wells. We observed significant induction of CD107a, a marker of NK cell degranulation and surrogate for cytolytic activity, and IFN-γ upon culture with either SARS-CoV-2-infected or mock-infected A549-ACE2 cells (Figures 1F and 1G). Activation occurred primarily within the CD56^bright^ CD16^low^ subset, possibly due to IL-2 priming (Figures 1F and S2). We also found no significant differences in the expression of other phenotypic and functional markers on NK cells co-cultured with SARS-CoV-2-infected targets compared with those cultured with mock-infected cells (Figure S2). This suggests that, while healthy NK cells are unable to lyse SARS-CoV-2-infected cells, the presence of SARS-CoV-2-infected cells does not inhibit the NK cell response to bystander cells. Collectively, these results support a model in which a factor intrinsic to SARS-CoV-2-infected cells allows escape of NK cell killing.

### SARS-CoV-2 infection modulates expression of ligands involved in NK cell recognition

**Figure 2:**
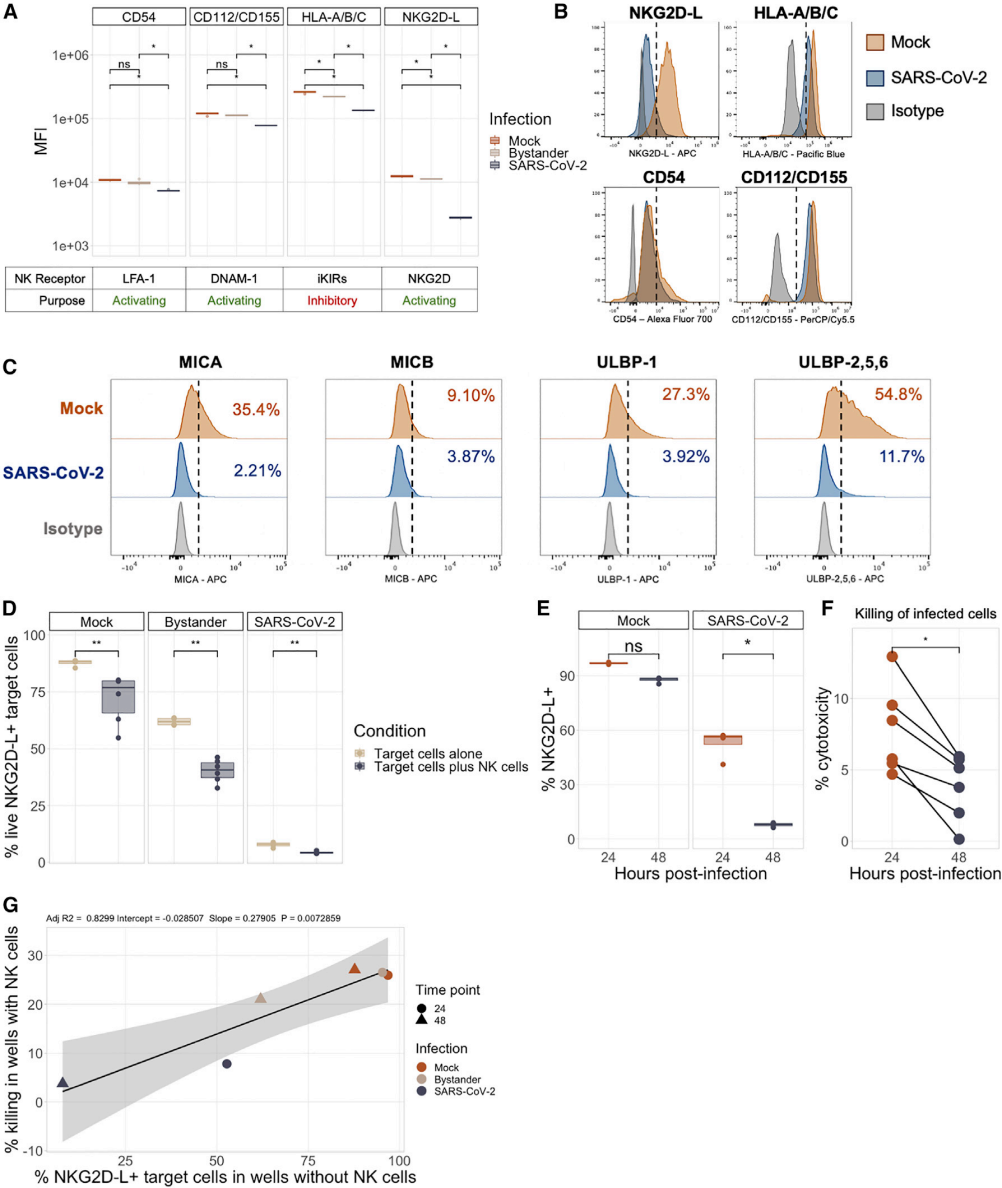
SARS-CoV-2 infection downregulates ligands for the activating receptor NKG2D (A) Boxplots showing the mean fluorescence intensity (MFI) of uninfected, bystander, and SARS-CoV-2-infected A549-ACE2 cells expressing CD54, CD112/CD155, HLA-ABC, and NKG2D-L (combination of MICA, MICB, and ULBPs 1, 2, 5, and 6). The cognate receptors recognizing each ligand are noted under each panel. Four technical replicates of each condition were performed. (B) Representative histograms of NKG2D-L, HLA-A/B/C, CD54, and CD112/CD155 expression in SARS-CoV-2-infected cells versus uninfected controls. Isotype controls are shown in gray. Vertical dashed lines represent thresholds for positivity. (C) Representative histograms showing expression of individual ligands for NKG2D in mock-infected (top) and SARS-CoV-2-infected (middle) A549-ACE2s. Isotype controls are shown at the bottom of each histogram for comparison. Dashed vertical lines represent thresholds for positivity. Numbers to the right of vertical lines indicate the percentage of cells positive for each marker. (D) Percentage of NKG2D-L-expressing A549-ACE2s in wells containing only target cells (n = 4 technical replicates) compared with wells containing target cells and NK cells (n = 6 biological replicates). Beginning at 48 h post-infection, target cells were co-cultured with IL-2-activated NK cells for 3 h at an E:T ratio of 5:1. (E) Percentage of mock-infected or SARS-CoV-2-infected (mNeonGreen+) A549-ACE2 expressing NKG2D-L at 24 and 48 h post-infection (n = 4 technical replicates per condition). (F) Background-subtracted target cell death of A549-ACE2 infected for either 24 or 48 h with SARS-CoV-2. Target cells were co-cultured for 3 h with IL-2-activated NK cells at an E:T ratio of 5:1. Lines connect data points from individual donors (n = 6). (G) Correlation between percentage of A549-ACE2s expressing NKG2D-L in target-only wells (mean of four technical replicates per condition) and background-subtracted target cell death in wells containing NK cells (mean of six biological replicates per condition). Significance values for (A, C, and D) were determined using an unpaired Wilcoxon ranked-sum test with the Bonferroni correction for multiple hypothesis testing. Significance value for (E) was determined using a paired Wilcoxon signed-rank test. Best-fit line shown in (F) was calculated using a linear model.

We next investigated the mechanism by which SARS-CoV-2-infected cells were able to evade lysis by NK cells. We used flow cytometry to profile the expression of the ligands for various NK cell activating and inhibitory receptors.^3^ We grouped antibodies for ligands recognized by the same receptor into a single channel to quantify total ligand density for a given receptor. While expression of CD112/CD155 (ligands for DNAM-1), CD54 (ligand for LFA-1), and HLA-A/B/C were decreased in infected cells compared with mock and bystander cells, the magnitude of these reductions was relatively small. In contrast, the ligands for NKG2D (MICA, MICB, and ULBPs 1, 2, 5, and 6; collectively referred to as NKG2D-L) were downregulated to a much greater extent in SARS-CoV-2-infected cells compared with uninfected cells and bystander cells (Figures 2A, 2B, and S4A). All of the individual ligands comprising NKG2D-L were strongly downregulated in SARS-CoV-2-infected cells compared with mock-infected controls (Figures 2C and S4B). Notably, the downregulation of NKG2D-L and the downregulation of HLA-A/B/C (MHC class I) would be expected to have opposing effects on the NK cell response to infected cells: downregulation of MHC class I would enhance NK cell recognition of infected targets, while NKG2D-L downregulation could represent a mechanism of NK cell evasion. As we observed a decrease in the ability of NK cells to kill SARS-CoV-2-infected cells and other studies have already interrogated MHC class I downregulation by SARS-CoV-2,^37^^,^^38^^,^^39^ we focused our attention on the loss of NKG2D-L as a potential evasion mechanism.

### Downregulation of NKG2D-L is correlated with inhibition of NK cell killing of SARS-CoV-2-infected cells

To evaluate the association between NKG2D-L expression and killing of SARS-CoV-2-infected cells, we assessed NKG2D-L expression on the cells that survived following co-culture with NK cells. We identified a significant decrease in the frequency of NKG2D-L-expressing target cells in wells containing NK cells at both time points and across all infection conditions, suggesting that NK cells preferentially kill NKG2D-L-expressing targets in both SARS-CoV-2-infected and mock-infected wells (Figure 2D). We also assessed the kinetics of NKG2D-L expression on infected (mNeonGreen+) A549-ACE2 and found that, while NKG2D-L were downregulated to some extent at 24 h post-infection compared with uninfected cells, it was not until 48 h post-infection that we observed almost total loss of these proteins at the surface level (Figure 2E). We therefore hypothesized that NK cells would kill infected cells more robustly at 24 h post-infection compared with 48 h. Indeed, we observed significantly better killing of mNeonGreen+ target cells at 24 h post-infection compared with 48 h (Figure 2F). Further supporting a model in which downregulation of NKG2D-L allows for evasion of NK cell killing, we identified a strong correlation between the expression of NKG2D-L in target cells and target cell lysis across all time points and infection conditions (Figure 2G).

### NK cells are able to efficiently kill SARS-CoV-2-infected cells immediately following infection

**Figure 3:**
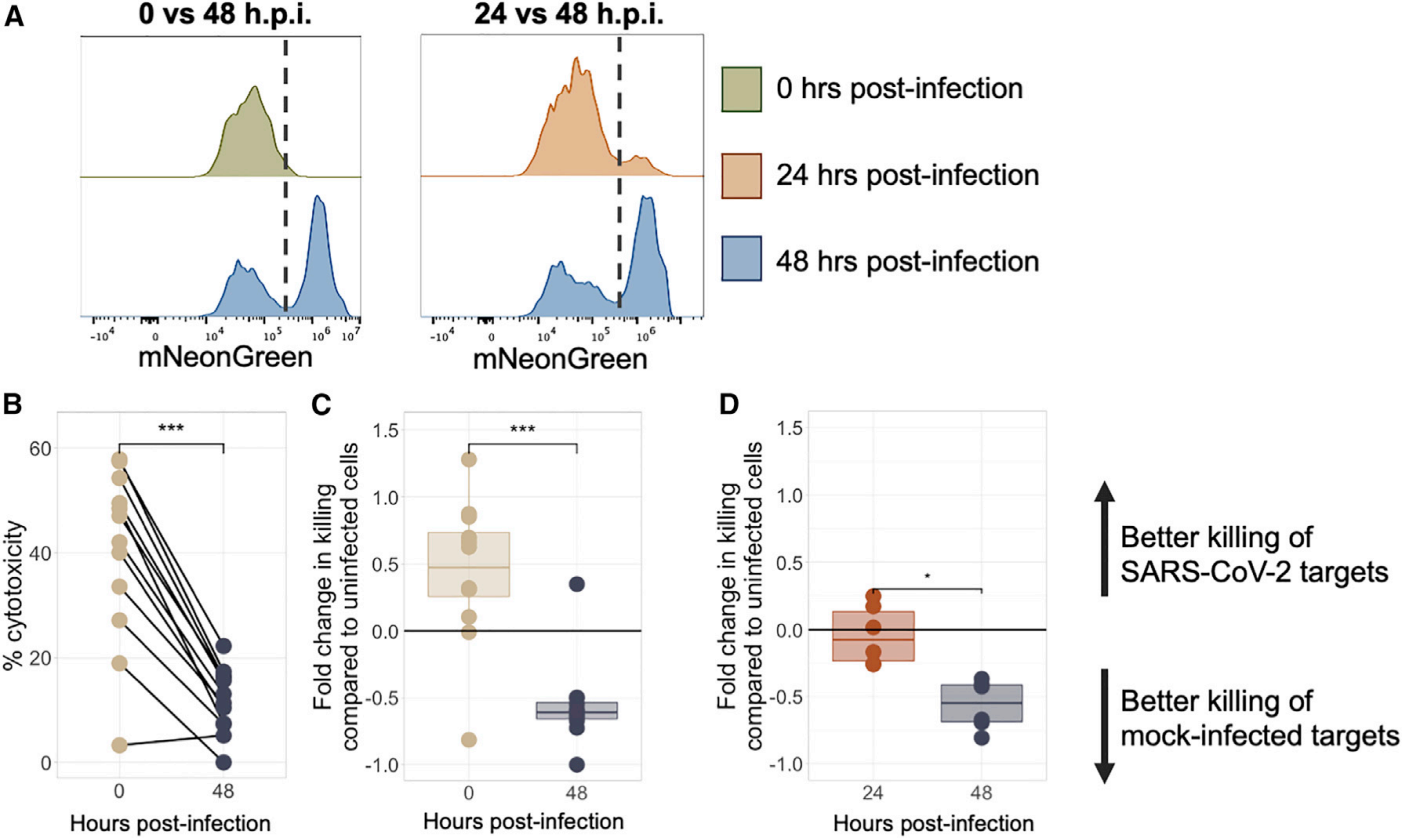
NK cells are able to efficiently kill SARS-CoV-2-infected cells immediately following infection (A) Representative histograms showing expression of mNeonGreen in SARS-CoV-2-exposed A549-ACE2 at 0, 24, or 48 h post-infection at an MOI of 3 (B and C) or 0.5 (D). Vertical dashed lines indicate threshold for positive gating. (B–D) A549-ACE2 were infected with SARS-CoV-2 for 0, 24, or 48 h, then co-cultured with IL-2-activated NK cells for 3 h at an E:T ratio of 5:1. (B) Background-subtracted killing of all single A549-ACE2s by NK cells following infection with SARS-CoV-2 for either 0 or 48 h. Lines connect points from individual donors (n = 12). (C) Fold change in killing of infected target cells compared with mock-infected target cells at 0 and 48 h post-infection. (D) Background-subtracted target cell death of all single A549-ACE2s infected with SARS-CoV-2 at 24 and 48 h post-infection (n = 6 unique donors). Significance values were determined using a paired Wilcoxon signed-rank test.

Other groups have reported that NK cells are able to successfully suppress viral replication in a system where the NK cells are added to a target cell culture soon after infection with SARS-CoV-2.^16^^,^^18^^,^^19^ Hammer et al. added NK cells to the co-culture immediately following infection; Witkowski et al. added NK cells 12 h post-infection; and Krämer et al. added NK cells 24 h post-infection. Given our finding that NKG2D-L are not fully downregulated until 48 h post-infection, we hypothesized that NK cells might be able to kill virus-infected cells in the early stages of infection, but not later. We therefore repeated our killing assay using infected or mock-infected cell cultures at either 0 h post-infection (similar to prior studies) or 48 h post-infection. Because the freshly infected cells had not yet expressed mNeonGreen at the time of analysis (Figure 3A), we compared total killing of all target cells in SARS-CoV-2-infected wells at 0 and 48 h. We found that, as expected, NK cells were able to robustly kill cells that were freshly infected (0 h) but not those that had been infected for 48 h (Figure 3B). Moreover, NK cells were slightly better at killing infected cells compared with mock-infected cells at the 0 h time point (Figure 3C), providing additional evidence that NK cells can successfully target infected cells in the early stages of SARS-CoV-2 infection, as previously reported.^16^^,^^18^^,^^19^ Finally, we conducted a similar analysis of total cell killing at 24 versus 48 h post-infection. In accordance with our other findings, we observed that NK cells can efficiently kill virus-exposed cells through 24 h post-infection, but not at 48 h (Figure 3D). Thus, our data and other published works collectively suggest that NK cells are capable of suppressing viral replication, but their ability to do so is significantly hampered if the cell has been infected for at least 48 h.

### SARS-CoV-2 protein Nsp1 downregulates ligands for NKG2D

**Figure 4:**
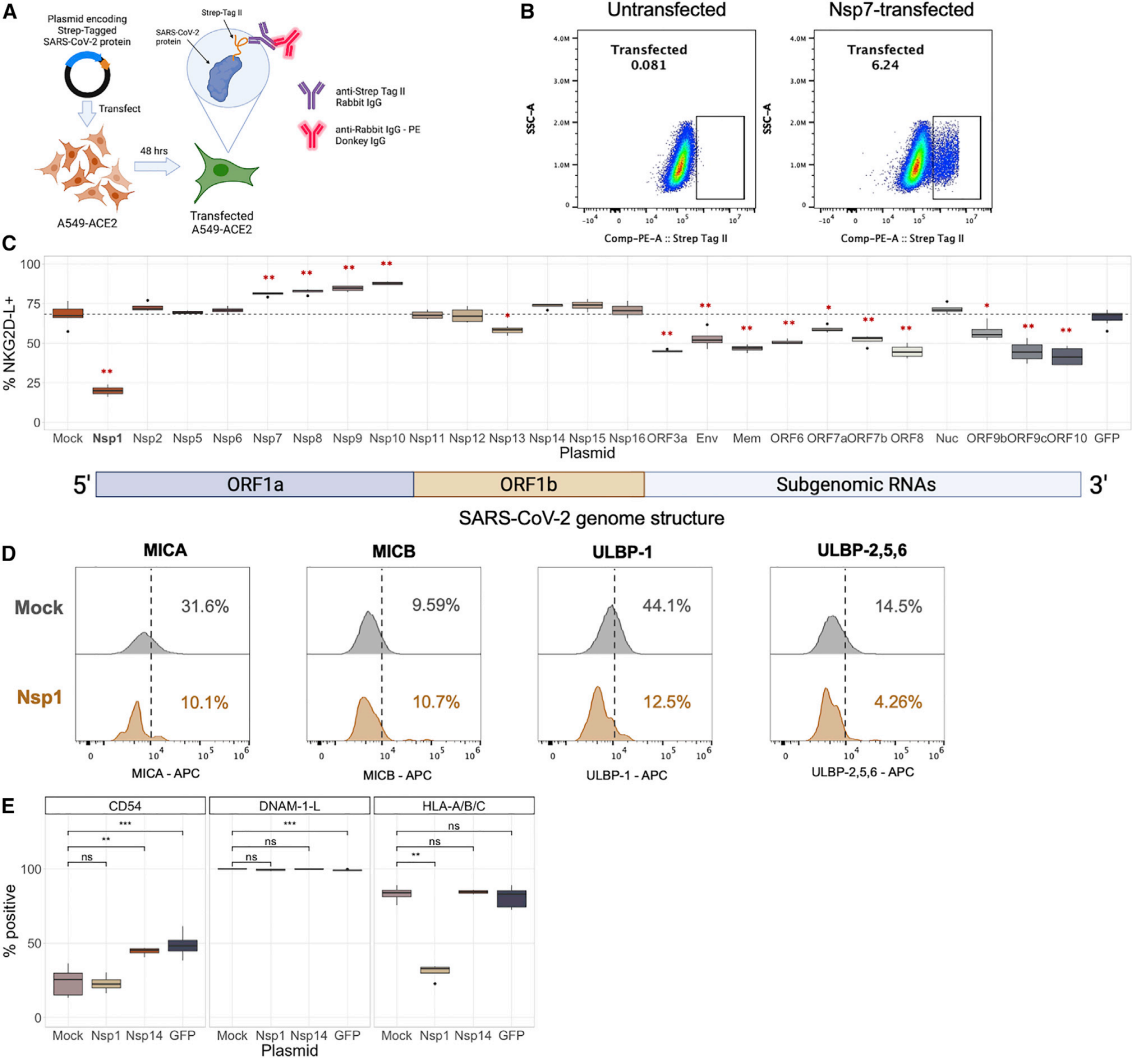
SARS-CoV-2 protein Nsp1 downregulates ligands for NKG2D (A) A schematic illustrating the experimental approach. Plasmids encoding individual SARS-CoV-2 proteins appended with two Strep-Tag domains were transfected into A549-ACE2s. After 48 h, transfected cells could be detected via flow cytometry using a primary antibody against Strep Tag II and a secondary fluorescent antibody. (B) Representative flow plots showing Strep Tag II expression in untransfected and Nsp7-transfected A549-ACE2s. (C) Percentage of transfected (Strep Tag II+) A549-ACE2s that express NKG2D-L by flow cytometry at 48 h post-transfection. 25 SARS-CoV-2 proteins are shown that were successfully transfected into A549-ACE2s, along with GFP as a transfection control. Four technical replicates were performed for each plasmid. Dashed line represents the mean frequency of expression in untransfected (mock) cells. Asterisks represent significance in comparison to mock-transfected controls. Plasmids are ordered by location in the SARS-CoV-2 genome and a schematic of the genome structure is shown below (C). (D) Representative histograms showing expression of individual ligands for NKG2D after transfection with transfection agent alone (top; mock) or Nsp1 (bottom). Dashed vertical lines indicate threshold for positivity. Numbers to the right of dashed lines show percentages of cells positive for each marker. (E) Percentage of A549-ACE2 positive for CD54, DNAM-1-L (CD112/CD155), or HLA-A/B/C at 48 h after transfection with transfection agent alone (mock), Nsp14, GFP, or Nsp1. Significance values for (C–E) were determined using an unpaired Wilcoxon rank-sum test with the Bonferroni correction for multiple hypothesis testing.

Having identified changes in the protein-level expression of NKG2D-L in SARS-CoV-2-infected cells that may underlie escape from NK cell killing, we next sought to understand how the virus mediates this effect. SARS-CoV-2 encodes 29 individual proteins that are broadly classified into 3 categories: structural, non-structural, and accessory. While the roles of these proteins are still being investigated, many of the non-structural and accessory proteins are known to suppress antiviral innate immune responses.^40^^,^^41^^,^^42^^,^^43^ We therefore transfected each individual SARS-CoV-2 protein, tagged with two Strep Tag domains (Strep Tag II) to allow for easy detection, into A549-ACE2 cells and assessed for their effect on NK cell receptor ligand expression by flow cytometry (Figures 4A and 4B). We successfully transfected 25 of the 29 SARS-CoV-2 proteins into A549-ACE2s; we also transfected cells with GFP as a non-viral control (Figures S5A–S5C). While several proteins downregulated NKG2D-L, SARS-CoV-2 non-structural protein 1 (Nsp1) had by far the strongest effect (Figures 4C and S5D). Several other viral proteins, primarily accessory proteins, also downregulated NKG2D-L expression, and some increased expression. However, as Nsp1 had the most impact on NKG2D-L expression, we chose to move forward with interrogation of this protein.

Like replication-competent SARS-CoV-2, Nsp1 also downregulated MICA, ULBP-1, and ULBPs-2, 5, and 6. However, it had no effect on MICB (Figure 4D). To ensure that the downregulation of NKG2D-L that we observed was not an artifact of the cell line we were using, we also transfected Nsp1 into 293T cells and K562 cells. Nsp1 downregulated NKG2D-L expression in both cell lines, which express NKG2D-L at baseline (Figure S6A). Nsp1 also mediated downregulation of MHC class I, but not CD54 or the ligands for DNAM-1, in A549-ACE2s (Figures 4F and S6B–S6D).

### SARS-CoV-2 post-transcriptionally downregulates NKG2D-L and does not induce shedding, intracellular retention, or degradation

Nsp1, also known as the SARS-CoV-2 leader protein, is the first protein translated when the virus enters a cell and serves as a global inhibitor of host translation. Nsp1 is highly conserved across coronaviruses as it plays an important role in enhancing pathogenicity by inhibiting the innate immune response.^44^^,^^45^^,^^46^^,^^47^^,^^48^^,^^49^ Schubert et al. demonstrated that SARS-CoV-2 Nsp1 functions by sterically inhibiting entry of mRNA into the mRNA channel of the 40S ribosomal subunit.^49^ Thus, it is likely that Nsp1 mediates a translational block to reduce surface NKG2D-L expression.

**Figure 5:**
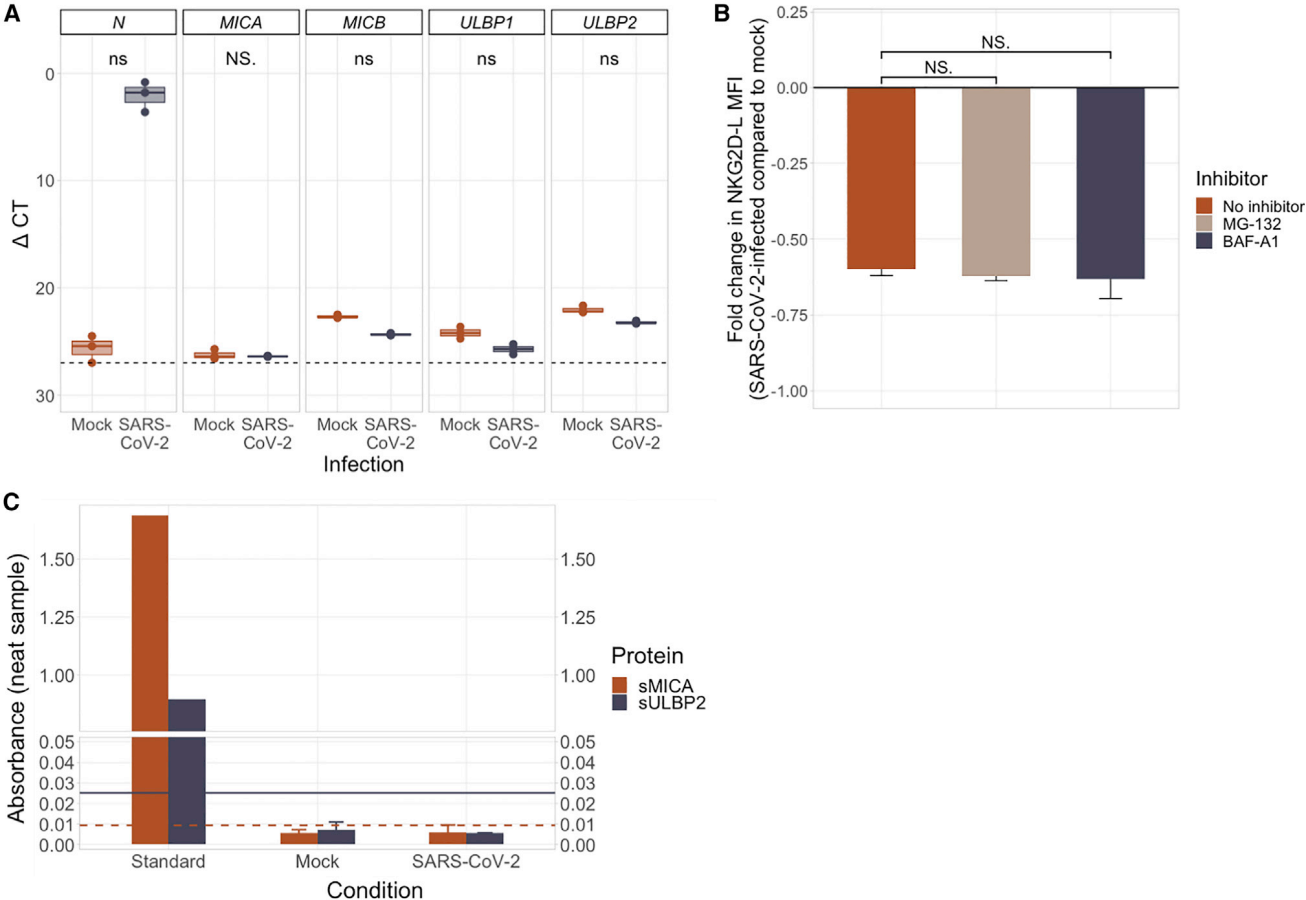
SARS-CoV-2 post-transcriptionally downregulates NKG2D-L and does not induce shedding or degradation (A) Boxplots showing the delta CT values of several genes in mock and SARS-CoV-2-infected A549-ACE2s as measured by qRT-PCR. N encodes SARS-CoV-2 nucleoprotein. MICA, MICB, ULBP1, and ULBP2 encode ligands for NKG2D. Each point represents the mean of three qPCR technical replicates. (B) Bar plot showing the fold change in mean fluorescence intensity (MFI) of NKG2D-L in SARS-CoV-2-infected A549-ACE2 compared with mock-infected cells after treatment with PBS (left), proteasome inhibitor MG-132 (middle), or lysosomal inhibitor BAF-A1 (right). Inhibitors were added 24 h after infection and NKG2D-L expression was measured by flow cytometry at 48 h post-infection. Bar plots represent mean values of three technical replicates ± standard deviations. (C) Absorbance values of neat supernatants from mock or SARS-CoV-2-infected cultures at varying dilutions as measured by plate-based ELISAs for soluble MICA (sMICA) and soluble ULBP2 (sUPBP2). Absorbance values were calculated by subtracting absorbance readings taken at 560 nm from those taken at 450 in accordance with the manufacturer’s instructions. Horizontal lines indicate limits of detection (dashed, sMICA; solid, sULBP2). Bar plots represent the means of four technical replicates for each condition ± standard deviations. Significance values in (A and B) were calculated using a Wilcoxon signed-rank test with the Bonferroni correction for multiple hypothesis testing.

To orthogonally validate that NKG2D-L expression is reduced via translational blockade in SARS-CoV-2-infected cells, we assessed several other potential methods of downregulation. Consistent with a model of translational inhibition, we observed only a small decrease in transcripts encoding MICB, ULBP-1, and ULBP-2 in infected cells compared with mock-infected cells and no decrease in *MICA* transcript levels (Figure 5A). This modest difference likely reflects the overall decrease in transcript levels in cells infected with SARS-CoV-2 and is consistent with the idea that NKG2D-L expression is reduced at the post-transcriptional level. We also assessed whether SARS-CoV-2 might induce degradation of NKG2D-L, as CMV has also been shown to downregulate NKG2D-L through targeting these proteins for proteasomal or lysosomal degradation.^31^^,^^32^ We therefore treated mock or SARS-CoV-2-infected cells with a proteasomal inhibitor (MG-132) or a lysosomal inhibitor (BAF-A1) and then assessed NKG2D-L expression; we found that neither inhibitor rescued NKG2D-L expression in infected cells (Figures 5B and S8A). Finally, we addressed the possibility of SARS-CoV-2-infected cells shedding of NKG2D-L from the cell surface, which has been reported for other viruses and in the setting of cancer,^24^^,^^50^^,^^51^ by assessing NKG2D-L levels in the supernatants of mock- and SARS-CoV-2-infected cultures by ELISA. We quantified levels of soluble MICA and soluble ULBP-2 (Figure 5C) as these were the two most highly expressed NKG2D ligands on mock-infected cells (Figure 2C). We were unable to detect either of these proteins in the supernatants of uninfected or infected cultures, suggesting that secretion of NKG2D ligands is not a major mechanism by which NKG2D-L is downregulated by SARS-CoV-2 (Figure 5C). Collectively, these data suggest that NKG2D-L are downregulated post-transcriptionally and are not degraded or shed in SARS-Cov-2-infected cells. While this supports the hypothesis that Nsp1 inhibits expression of these proteins by translational blockade, we were unable to definitively prove this mechanism, as expression of NKG2D-L could be suppressed by another mechanism such as intracellular retention.^23^^,^^29^

### NKG2D-L have a high rate of surface turnover

Although Nsp1 is a global inhibitor of host translation, our data show that it does not equally downregulate all NK cell receptor ligands. We hypothesized that this might be due to differential rates of surface expression turnover across the various ligands, as these proteins are known to have varying levels of stability on the cell surface.^32^^,^^52^^,^^53^^,^^54^ NKG2D-L in particular are rapidly turned over to allow for a high degree of control over its expression level.^32^^,^^52^ To validate that non-specific inhibition of a post-transcriptional mechanism could have an outsized effect on NKG2D-L in comparison with the other ligands assayed, we treated A549-ACE2s with the protein transport inhibitor Brefeldin A and measured expression of NK cell receptor ligands after 24 or 48 h (Figure S7). We observed that Brefeldin A, like Nsp1, had a much larger effect on NKG2D-L than on other ligands, including CD54 and DNAM-1 ligands, supporting a model in which global translation inhibition, such as that mediated by Nsp1, could much more dramatically downregulate NKG2D-L than other surface proteins.

### Nsp1 is not highly expressed until more than 24 h post-infection

**Figure 6:**
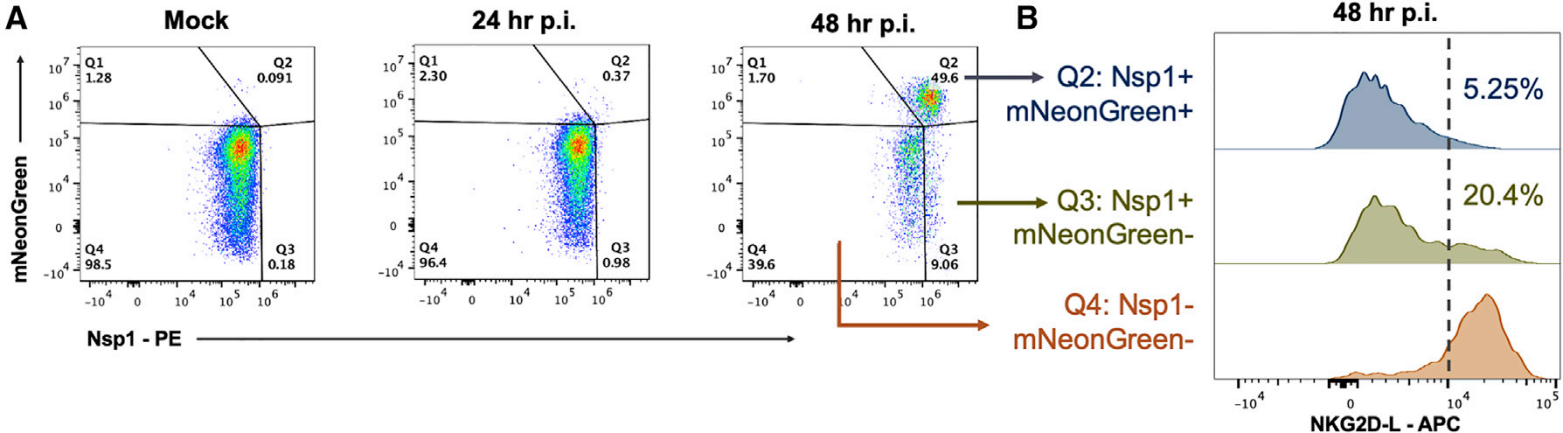
Nsp1 is not highly expressed in SARS-CoV-2-infected cells until >24 h post-infection and negatively correlates with NKG2D-L (A) Representative flow plots showing expression of Nsp1 and mNeonGreen in mock-infected A549-ACE2 (left), A549-ACE2 infected with SARS-CoV-2 for 24 h (middle), or A549-ACE2 infected with SARS-CoV-2 for 48 h (right). (B) Representative histograms showing NKG2D-L expression across subpopulations of SARS-CoV-2-infected cells at 48 h post-infection. Dashed vertical line indicates threshold for positivity. Numbers to the right of the dashed vertical line represent the percentage of cells positive. These data are representative of three technical replicates for each condition.

Thus far, our analyses of NK cell evasion mediated by replication-competent SARS-CoV-2 have relied on mNeonGreen as a correlate of viral protein expression. However, having determined that Nsp1 is the viral protein with the strongest effect on NKG2D-L expression, we wanted to validate (1) that mNeonGreen expression correlates with Nsp1 expression and (2) that Nsp1 expression inversely correlates with NKG2D-L expression in SARS-CoV-2-infected cells. We therefore stained SARS-CoV-2-infected or mock-infected A549-ACE2s with an anti-Nsp1 antibody and compared expression of Nsp1 to expression of mNeonGreen by flow cytometry. We found that essentially all mNeonGreen+ cells also expressed Nsp1 (Figure 6A). In addition, we determined that, like mNeonGreen, we could not detect high levels of Nsp1 expression until >24 h post-infection (Figure 6A); this aligns with our data demonstrating that SARS-CoV-2-infected cells are not fully resistant to NK cell killing until >24 h post-infection (Figures 2E and 2F).

While all mNeonGreen+ cells also expressed Nsp1, there was a significant population of cells (∼10%) at 48 h post-infection that expressed Nsp1 but not mNeonGreen (Figure 6A). This can likely be explained by the fact that Nsp1 is encoded at the 5′-most end of the SARS-CoV-2 genome and is thus the first viral protein to be translated.^44^^,^^45^^,^^46^^,^^47^^,^^48^^,^^49^ This suggests that identification of infected cells based solely on mNeonGreen expression slightly underestimates the number of infected cells and likely explains why bystander cells appear to have slightly decreased expression of NKG2D-L compared with mock-infected cells; the bystander population includes some cells that have been recently infected and express Nsp1 but not yet mNeonGreen. It also allowed us to assess expression of NKG2D-L-infected cells subsetted by their expression of mNeonGreen and Nsp1. As expected, Nsp1− mNeonGreen− (Q4) cells had high expression of NKG2D-L, while Nsp1+ mNeonGreen+ (Q2) cells had lost almost all expression of NKG2D-L. However, Nsp1+ mNeonGreen– (Q3) cells had an intermediate level of NKG2D-L expression, with roughly 20% of this population expressing these ligands (Figure 6B). We hypothesize that these cells are more recently infected and have not yet expressed the full complement of viral proteins. Therefore, these data suggest that NKG2D-L downregulation precedes expression of at least some viral proteins.

### Nsp1 is sufficient to confer significant resistance to NK cell-mediated killing

**Figure 7:**
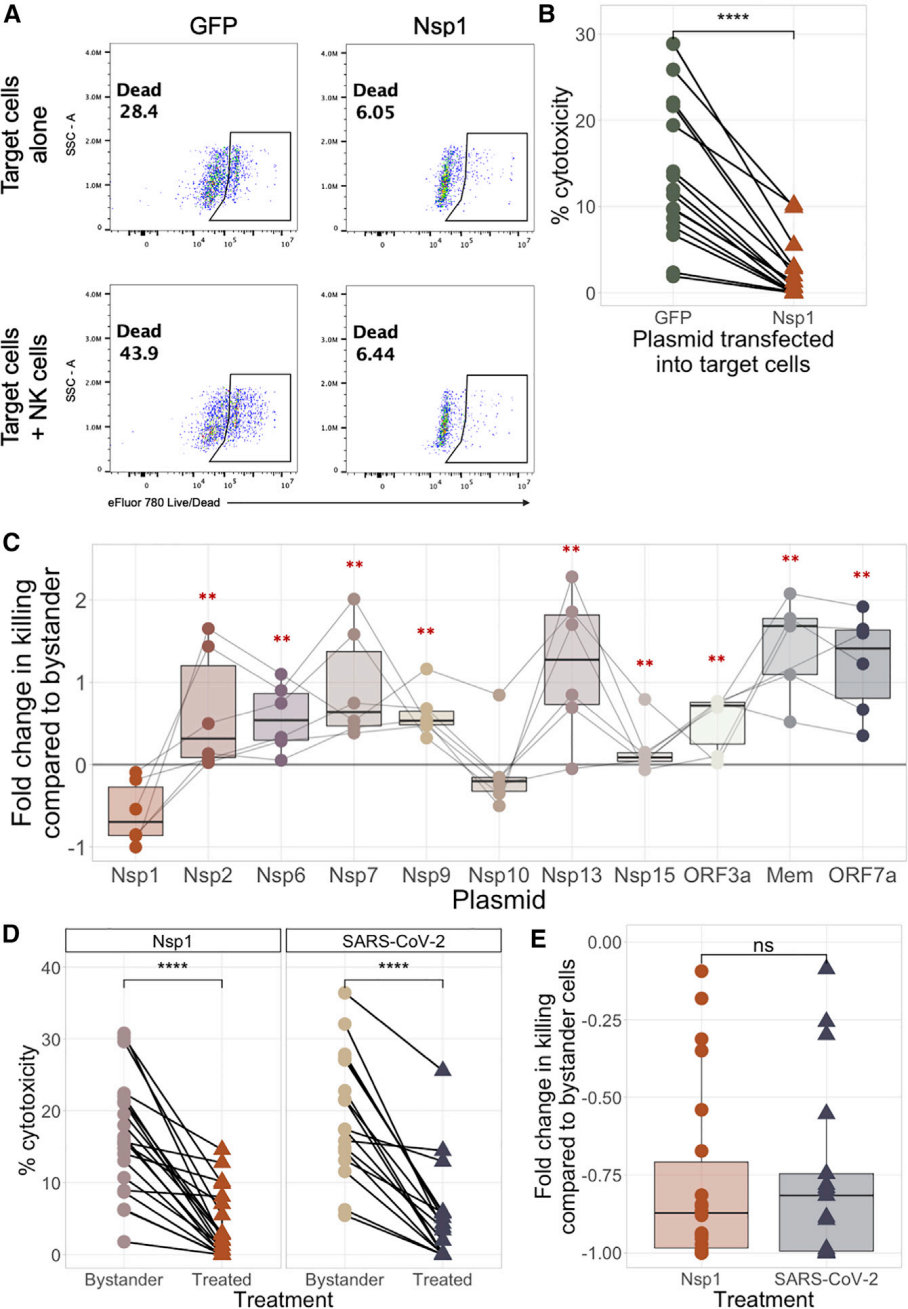
Nsp1 is sufficient to confer significant resistance to NK cell-mediated killing (A) Representative flow plots showing expression of eFluor 780 viability dye in target cells with NK cells (top) and with NK cells (bottom). (B) Background-subtracted target cell death among cells transfected with either GFP or Nsp1 following co-culture with healthy NK cells (E:T = 5:1) for 3 h (n = 22 unique donors). (C) Fold change in killing of target cells transfected with various SARS-CoV-2 proteins compared with untransfected bystander cells. The same healthy NK cell donors were utilized for all killing assays performed in (C). Asterisks represent significance in comparison to Nsp1-transfected cells. Transfected cells were gated by Strep Tag II expression before the percentage of cytotoxicity was determined. n = 6 unique donors. (D) Background-subtracted target cell death in treated (transfected with Nsp1 or infected with SARS-CoV-2) versus bystander A549-ACE2s following co-culture with healthy NK cells (E:T = 5:1) for 3 h. Lines in (B–D) represent individual donors. (E) Boxplot quantifying the fold change in background-subtracted target cell death between bystander (uninfected/untransfected) cells and cells that were positive for either Nsp1 (transfected; n = 22) or SARS-CoV-2 (infected; n = 18). Significance values were determined using a paired Wilcoxon signed-rank test (B–D) or unpaired Wilcoxon ranked-sum test (E) with the Bonferroni correction for multiple hypothesis testing where necessary.

We hypothesized that, if Nsp1 is the key mediator of NKG2D-L downregulation in SARS-CoV-2 infection, transfection with Nsp1 should be sufficient to confer resistance to NK cell killing. To test this hypothesis, we co-cultured activated, healthy NK cells with cells that had been transfected with either Nsp1 or a control plasmid (GFP) and assessed target cell killing by flow cytometry. Indeed, we found that NK cells were significantly more effective at killing GFP-transfected targets compared with Nsp1-transfected targets in both A549-ACE2s and 293Ts (Figures 7A, 7B, and S8). To determine whether other viral proteins might also mediate escape from NK cell killing, we compared killing of Nsp1-transfected target cells with killing of cells transfected with other SARS-CoV-2 proteins (Figures 7C and S8). We randomly selected 10 additional SARS-CoV-2 proteins to test alongside Nsp1. Each protein was transfected into A549-ACE2s and healthy NK cell killing of transfected cells was assessed 48 h post-transfection. We distinguished transfected cells from untransfected cells within the same well by gating on Strep Tag II expression. Of the 11 proteins transfected, Nsp1-transfected cells were killed significantly less than those transfected with any other plasmid except Nsp10 (no significant difference) (Figure 7C). Nsp1 was also the only protein that significantly protected transfected cells from NK cell killing (Figures 7C and S8D). Moreover, 6 of the other 10 proteins tested caused a significant *increase* in NK cell killing of transfected cells (Figure S8D). Collectively, these data suggest that Nsp1 is sufficient to protect cells from NK-mediated killing and that resistance to NK cell killing in infected cells overcomes the increase in susceptibility to killing caused by other SARS-CoV-2 proteins.

Finally, we sought to compare the protection from NK cell killing mediated by Nsp1 transfection to that conferred by infection with replication-competent SARS-CoV-2. Like SARS-CoV-2, Nsp1 was able to provide significant protection to cells that received the protein versus bystander cells in the same well (Figure 7D). We then quantified protection from killing by calculating the fold change in killing of treated (Nsp1-transfected or SARS-CoV-2-infected) compared with bystander cells for each donor and found that there was no significant difference between the level of protection mediated by Nsp1 and that mediated by SARS-CoV-2 (Figure 7E).

## Discussion

The role of NK cells in mediating clearance of SARS-CoV-2-infected cells *in vivo* remains unclear. While several studies have demonstrated that NK cells can reduce the levels of SARS-CoV-2 replication *in vitro*, no prior study has directly evaluated killing of SARS-CoV-2-infected cells. Here, we address this critical gap in knowledge and demonstrate that SARS-CoV-2-infected cells escape killing by healthy NK cells in a cell-intrinsic manner, while killing of uninfected bystander cells is uninhibited. The ability of infected cells to evade NK cell recognition requires infection to proceed long enough to allow an infected cell to express SARS-CoV-2-encoded proteins. We demonstrate that this escape mechanism is driven by downregulation of ligands for NKG2D, a critical activating receptor on NK cells. We further demonstrate that this ligand downregulation is driven by the SARS-CoV-2 Nsp1 protein and show that Nsp1 alone is sufficient to mediate direct NK cell evasion. While our experimental system using a cell line with high expression of NKG2D-L could enhance the degree of bystander killing, these findings have important implications for NK cell-mediated control of SARS-CoV-2, as preferential escape of infected cells and possible killing of bystander cells could contribute to SARS-CoV-2 pathogenesis.

These results illustrate the importance of examining the temporal dynamics of the NK cell response to SARS-CoV-2-infected cells. Other studies have assessed the ability of NK cells to suppress viral load by co-culturing NK cells with SARS-CoV-2-infected targets early after infection; their results suggest that, under these conditions, NK cells can at least partially control viral replication.^16^^,^^18^^,^^19^ It is worth noting that these other studies also varied from ours in parameters such as target cell type, cytokine treatment of NK cells, E:T ratio, and duration of co-culture. Our own observations demonstrate that NK cells are no longer able to effectively kill infected cells when added to the culture at 48 h post-infection, after the expression of viral proteins that suppress the innate immune response. The preferential killing of NKG2D-L-positive bystander cells may have important implications for lung pathology during COVID-19. NKG2D-L can be expressed by most cell types^55^ and are upregulated during viral infections, including HIV^56^ and RSV,^57^ in response to stress.^58^ Therefore, it is possible that NK cells may actually cause damage to the healthy tissue surrounding infected cells rather than clearing the infection, although this hypothesis has not yet been directly tested in primary lung tissue. As NK cells appear to home to the lungs during COVID-19,^59^^,^^60^^,^^61^ our findings indicate that the timing of NK cell trafficking to the site of infection may impact the efficacy of the NK cell response to SARS-CoV-2 infection, as there is a very narrow window for killing of infected cells before bystander killing could ensue. Interestingly, Witkowski et al. observed that frequency of peripheral blood NK cells in severe COVID-19 patients negatively correlated with viral load; however, this is difficult to interpret in the context of our data because it is unknown whether the increased NK cell frequencies observed resulted from decreased trafficking to the lungs, increased peripheral proliferation, or another mechanism.^19^

Our novel finding that the SARS-CoV-2 protein Nsp1 mediates evasion of NK cell killing has significant implications for both the study of the immune response to coronaviruses and the development of therapeutics for COVID-19. Nsp1 is highly conserved across coronaviruses and is an essential virulence factor; it has been shown to inhibit translation of host antiviral factors across multiple beta-coronaviruses.^44^^,^^45^^,^^46^^,^^47^^,^^48^^,^^49^^,^^62^ One study found that, among nearly 50,000 SARS-CoV-2 sequences analyzed, only 2.4% had any mutations within Nsp1.^46^ SARS-CoV-2 Nsp1 also shares 84.4% of its sequence identity with SARS-CoV Nsp1. Moreover, critical motifs within Nsp1 involved in the inhibition of innate immune responses are highly conserved across many beta-coronaviruses.^46^ On a practical level, the high degree of conservation of Nsp1 and its importance in coronavirus virulence have already made this protein the focus of several therapeutic strategies.^44^^,^^63^^,^^64^ Our work demonstrates that Nsp1 is an even more attractive target than previously thought, as inhibiting the function of this protein has the potential to fully or partially rescue the NK cell response to SARS-CoV-2-infected cells.

Although Nsp1 is a global inhibitor of host translation, our data demonstrate that it has an outsized effect on NKG2D-L and MHC class I surface expression compared with that of other ligands for NK cell receptors. This appears to be due to the varying stabilities of the different ligands on the cell surface, rather than explicit specificity of Nsp1 for NKG2D-L or MHC class I. It has been established that NKG2D-L are rapidly turned over on the cell surface and are quickly lost upon treatment with a protein transport inhibitor such as Brefeldin A.^32^^,^^52^ MHC class I is similarly transient on the cell surface in the presence of translation inhibition, although its stability varies with haplotype and peptide binding.^53^ CD54, which was not affected by Nsp1, is highly stable for at least 48 h, even after treatment with similar inhibitors.^54^ Thus, the differential effects of Nsp1 on various ligands for NK cell receptors are likely explained by the varying kinetics of surface turnover.

One of our findings that has been demonstrated by multiple groups is the downregulation of MHC class I upon SARS-CoV-2 infection. The mechanism of this downregulation remains unclear; while our data suggest that Nsp1 is responsible for this loss, ORF3a,^37^ ORF7a,^37^ ORF6,^38^ and ORF8^39^ have also been implicated. This could be due to differential downregulation of various HLA molecules by different SARS-CoV-2 proteins. In our study, we grouped together HLAs A, B, and C as there are no commercially available antibody clones that can robustly differentiate HLAs A and B; this is an important limitation of our work. According to the well-established “missing self” model of NK cell activation,^65^^,^^66^ the downregulation of self-MHC can induce NK cell activation through subsequent lack of inhibitory signaling through the killer cell immunoglobulin-like receptors. Therefore, it might be expected that the downregulation of MHC by SARS-CoV-2 would enhance the ability of NK cells to lyse infected cells—precisely the opposite of what we observed in our study. We hypothesize that this can be explained by (1) the relative magnitudes of MHC class I and NKG2D-L downregulation on infected cells and (2) the accepted dogma in the field that missing self alone is not sufficient to cause robust NK cell activation.^67^^,^^68^ As a result, we propose that the loss of NKG2D-L is the dominant factor in the NK cell response (or lack thereof) to SARS-CoV-2.

While our study focuses on direct lysis of target cells, NK cells can also kill through antibody-dependent cellular cytotoxicity. A recent study by Fielding et al. found that antibody-dependent NK cell activation can overcome SARS-CoV-2’s inhibition of direct cytotoxicity, allowing healthy NK cells to mount stronger responses to infected targets. Thus, prior vaccination or infection that results in pre-existing antibodies to SARS-CoV-2 could tip the balance in favor of killing SARS-CoV-2-infected cells. This study also identified downregulation of NKG2D-L on SARS-CoV-2-infected cells through an orthogonal method.^20^

This work has significant implications for the ongoing study of COVID-19. Our results deeply interrogate a potential flaw in the ability of the immune system to mount a comprehensive immune response to COVID-19. We demonstrate that the timing of the NK cell response to SARS-CoV-2-infected target cells is critical, with NK cells being able to control viral replication early in infection, but not after expression of viral proteins has begun. This should be further interrogated *in vivo* to explore whether the kinetics of NK cell trafficking during COVID-19 affects disease outcome. Finally, we reveal that SARS-CoV-2 protein Nsp1 is a major factor in mediating evasion of NK cell killing. This finding reinforces the attractiveness of Nsp1 as a therapeutic target.

### Limitations of the study

Our study has several limitations. To focus on NK cell responses in the respiratory tract, we used A549-ACE2 cells, which are an immortalized, malignant cell line. This could therefore have enhanced NK cell targeting of bystander cells. In addition, while we demonstrated that Nsp1 was sufficient to confer NK cell escape, we were unable to test whether the absence of Nsp1 rescues NK cell killing because knockout of Nsp1 is lethal to the virus. We also did not fully evaluate why Nsp1 blocks NKG2D-L more effectively than other proteins, but we hypothesize that these proteins are downregulated first as part of the global translation block because they are turned over on the cell surface more quickly and cannot be replaced. Finally, we did not interrogate the ability of every individual SARS-CoV-2 protein to mediate escape from NK cell killing.

## STAR★Methods

### Key resources table

**Table:** 

REAGENT or RESOURCE	SOURCE	IDENTIFIER
**Antibodies**		

See Table S1 for list of antibodies and other flow cytometry reagents used in this study.		N/A

**Bacterial and virus strains**		

icSARS-CoV-2/WA-01-mNeonGreen	Lab of Dr. Pei-Yong Shi	https://www.ncbi.nlm.nih.gov/pmc/articles/PMC7153529/

**Chemicals, peptides, and recombinant proteins**		

DNA/RNA Shield	Zymo Research	Cat#R1100-250
16% paraformaldehyde	Electron Microscopy Sciences	Cat#15710
RNA Clean & Concentrator Kit	Zymo Research	Cat#R1018
TURBO DNA-free Kit	Fisher Scientific	Cat#AM1907
Brefeldin A	eBioscience	Cat#00-4506-51
Monensin	eBioscience	Cat#00-4505-51
10x Permeabilization Buffer	BD Biosciences	Cat#340973
rhIL-2	R&D Systems	Cat#202-IL-010
MACS Human NK Cell Isolation Kit	Miltenyi	Cat#130-092-657
PBS	ThermoFisher Scientific	Cat#10010023
DMEM	Life Technologies	Cat#11885–092
TrypLE	ThermoFisher Scientific	Cat#12604021
MG-132	abcam	Cat#ab141003
BAF-A1	Millipore Sigma	Cat#SML1661-.1ML
Fugene HD	Promega	Cat#E2311
Lipofectamine 3000	ThermoFisher Scientific	Cat#L3000001
Triton-X 100	Sigma-Aldrich	Cat#T9284-100ML

**Critical commercial assays**		

Invitrogen superscript III Platinum One Step qRT PCR Kit with ROX	Invitrogen	Cat#11745500
Human MICA DuoSet ELISA	R&D Systems	Cat#DY1800
Human ULBP-2 DuoSet ELISA	R&D Systems	Cat#DY1298

**Experimental models: Cell lines**		

Vero E6 cell line	ATCC	Cat#CRL-1586
K562 cell line	ATCC	Cat#CCL-243
293T cell line	ATCC	Cat#CRL-3216
CEM.NKR	HIV Reagent Program	Cat#ARP-5198
A549-ACEs	Lab of Dr. Ralf Bartenschlager	

**Oligonucleotides**		

*MICA* RT-qPCR primer-probe kit	ThermoFisher Scientific	Assay ID: Hs07292198_gH
*MICB* RT-qPCR primer-probe kit	ThermoFisher Scientific	Assay ID: Hs00792952_m1
*ULBP1* RT-qPCR primer-probe kit	ThermoFisher Scientific	Assay ID: Hs0036941_m1
*ULBP2* RT-qPCR primer-probe kit	ThermoFisher Scientific	Assay ID: Hs01127964_m1
SARS-CoV-2 *N* RT-qPCR fwd primer	Biosearch Technologies	Cat#nCoV-N1-F-100
SARS-CoV-2 *N* RT-qPCR rev primer	Biosearch Technologies	Cat#nCoV-N1-R-100
SARS-CoV-2 *N* RT-qPCR probe	Biosearch Technologies	Cat#nCoV-N1-P-25
*18S* control RT-qPCR primer-probe kit	ThermoFisher Scientific	Cat#4352930E

**Recombinant DNA**		

pLVX-EF1alpha-SARS-CoV-2-nsp1-2xStrep-IRES-Puro	Lei S. Qi Lab, Stanford University	Addgene Plasmid #141367
pLVX-EF1alpha-SARS-CoV-2-nsp2-2xStrep-IRES-Puro	Lei S. Qi Lab, Stanford University	Addgene Plasmid #141368
pLVX-EF1alpha-SARS-CoV-2-nsp5-2xStrep-IRES-Puro	Lei S. Qi Lab, Stanford University	Addgene Plasmid #141371
pLVX-EF1alpha-SARS-CoV-2-nsp6-2xStrep-IRES-Puro	Lei S. Qi Lab, Stanford University	Addgene Plasmid #141372
pLVX-EF1alpha-SARS-CoV-2-nsp7-2xStrep-IRES-Puro	Lei S. Qi Lab, Stanford University	Addgene Plasmid #141373
pLVX-EF1alpha-SARS-CoV-2-nsp8-2xStrep-IRES-Puro	Lei S. Qi Lab, Stanford University	Addgene Plasmid #141374
pLVX-EF1alpha-SARS-CoV-2-nsp9-2xStrep-IRES-Puro	Lei S. Qi Lab, Stanford University	Addgene Plasmid #141375
pLVX-EF1alpha-SARS-CoV-2-nsp10-2xStrep-IRES-Puro	Lei S. Qi Lab, Stanford University	Addgene Plasmid #141376
pLVX-EF1alpha-SARS-CoV-2-nsp11-2xStrep-IRES-Puro	Lei S. Qi Lab, Stanford University	Addgene Plasmid #141377
pLVX-EF1alpha-SARS-CoV-2-nsp12-2xStrep-IRES-Puro	Lei S. Qi Lab, Stanford University	Addgene Plasmid #141378
pLVX-EF1alpha-SARS-CoV-2-nsp13-2xStrep-IRES-Puro	Lei S. Qi Lab, Stanford University	Addgene Plasmid #141379
pLVX-EF1alpha-SARS-CoV-2-nsp14-2xStrep-IRES-Puro	Lei S. Qi Lab, Stanford University	Addgene Plasmid #141380
pLVX-EF1alpha-SARS-CoV-2-nsp15-2xStrep-IRES-Puro	Lei S. Qi Lab, Stanford University	Addgene Plasmid #141381
pLVX-EF1alpha-SARS-CoV-2-nsp16-2xStrep-IRES-Puro	Lei S. Qi Lab, Stanford University	Addgene Plasmid #141382
pLVX-EF1alpha-SARS-CoV-2-orf3a-2xStrep-IRES-Puro	Lei S. Qi Lab, Stanford University	Addgene Plasmid #141383
pLVX-EF1alpha-SARS-CoV-2-orf6-2xStrep-IRES-Puro	Lei S. Qi Lab, Stanford University	Addgene Plasmid #141387
pLVX-EF1alpha-SARS-CoV-2-orf7a-2xStrep-IRES-Puro	Lei S. Qi Lab, Stanford University	Addgene Plasmid #141388
pLVX-EF1alpha-SARS-CoV-2-orf7b-2xStrep-IRES-Puro	Lei S. Qi Lab, Stanford University	Addgene Plasmid #141389
pLVX-EF1alpha-SARS-CoV-2-orf8-2xStrep-IRES-Puro	Lei S. Qi Lab, Stanford University	Addgene Plasmid #141390
pLVX-EF1alpha-SARS-CoV-2-orf9b-2xStrep-IRES-Puro	Lei S. Qi Lab, Stanford University	Addgene Plasmid #141392
pLVX-EF1alpha-SARS-CoV-2-orf9c-2xStrep-IRES-Puro	Lei S. Qi Lab, Stanford University	Addgene Plasmid #141393
pLVX-EF1alpha-SARS-CoV-2-orf10-2xStrep-IRES-Puro	Lei S. Qi Lab, Stanford University	Addgene Plasmid #141394
pLVX-EF1alpha-SARS-CoV-2-N-2xStrep-IRES-Puro	Lei S. Qi Lab, Stanford University	Addgene Plasmid #141391
pLVX-EF1alpha-SARS-CoV-2-E−2xStrep-IRES-Puro	Lei S. Qi Lab, Stanford University	Addgene Plasmid #141385
pLVX-EF1alpha-SARS-CoV-2-M-2xStrep-IRES-Puro	Lei S. Qi Lab, Stanford University	Addgene Plasmid #141386
pLVX-EF1alpha-eGFP-2xStrep-IRES-Puro	Lei S. Qi Lab, Stanford University	Addgene Plasmid #141395

**Software and algorithms**		

R Studio	https://www.rstudio.com/	https://www.rstudio.com/
FlowJo v10.7.1	https://www.flowjo.com/	https://www.flowjo.com/

### Resource availability

#### Lead contact

Inquiries, comments, and requests for additional information and/or data may be directed to the corresponding author, Dr. Catherine Blish (cblish@stanford.edu).

#### Materials availability

No new materials were generated by this study.

### Experimental model and subject details

#### Healthy donor PBMC

The primary immune cells used in this study were isolated from leukoreduction system (LRS) chambers obtained from anonymous, healthy donors through the Stanford Blood Bank. Age and sex information were not provided as all samples were obtained anonymously. PBMC were isolated by Ficoll-Paque PLUS (Millipore Sigma, Cat. GE17-1440-02) and cryopreserved in liquid nitrogen. All LRS chambers were collected prior to the start of the COVID-19 pandemic (Nov. 2019 or earlier); donors were thus naive to SARS-CoV-2 infection.

#### Cell lines

A549-ACE2s were a gift from Ralf Bartenschlager. VeroE6, 293T, K562, and CEM.NKR-CCR5 cells were obtained from ATCC. All cell lines were confirmed to be mycoplasma-free. A549-ACE2 cultures were replenished after no more than 25 passages to ensure integrity of ACE2 expression. All other cell lines were not maintained long-term in culture. When passaging A549-ACE2 cells, TrypLE (ThermoFisher, Cat. 12604021) was used instead of standard trypsin to preserve the integrity of cell surface proteins, including ACE2. All cell lines were cultured at 37°C, 5% CO_2_.

#### Viral stock generation and titration

icSARS-CoV-2/WA-01-mNeonGreen was a kind gift from Dr. Pei-Yong Shi. Virus was passaged twice in VeroE6 cells and titered by plaque assay on VeroE6 cells using Avicel (FMC Biopolymer) overlay. Passage 3 was used for all experiments. The viral stock was deep-sequenced and aligned to reference genomes in GenBank to confirm sequence.

### Method details

#### Infection with SARS-CoV-2

A549-ACE2 cells were plated at a density of 0.1e6 cells/mL the day prior to infection. On the day of infection, A549-ACE2s were washed with PBS (ThermoFisher Scientific, Cat. 10010023), placed in DMEM (Life Technologies, Cat. 11885–092) supplemented with 2% FBS (“D2”), and brought into the BSL3 laboratory. The D2 was removed and virus was added at an appropriate MOI (0.5 unless otherwise noted) in D2 in a volume equal to 53 uL/cm^2^ of culture surface area. Mock-infected cells received D2 containing no virus. The infected or mock-infected cells were rocked at 37°C for 1 hour, after which time they were washed with PBS to remove unbound virions. Fresh D2 was then added to the cells and they were replaced into a 37°C incubator for 0–48 hours.

#### NK cell isolation and activation

NK cells were isolated from cryopreserved healthy donor PBMC using the Miltenyi MACS Human NK Cell Isolation Kit (Miltenyi, Cat. 130-092-657) according to the manufacturer’s instructions. Following isolation, NK cells were transferred to a round-bottom 96-well plate and resuspended in complete RPMI supplemented with 25 ng/mL (250 IU/mL) rhIL-2 (R&D Systems, Cat. 202-IL-010). NK cells were then placed into a 37°C incubator. After 12–16 hours, the NK cells were washed twice to remove IL-2, counted, and plated in a fresh round-bottom 96-well plate for killing and/or functional assays.

#### Flow cytometry-based killing and NK cell activation assays

SARS-CoV-2-infected or mock-infected A549-ACE2 cells were harvested using TrypLE and counted. The cells were washed and transferred to complete RPMI, then added to the NK cell cultures at an E:T ratio of 5:1 for killing assays and 1:2 for NK cell activation assays. Additional wells containing only target cells or only NK cells were included for control purposes. For activation assays, CD107a-PE, Brefeldin A (eBioscience, Cat. 00-4506-51), and Monensin (eBioscience, Cat. 00-4505-51) were added to all wells at the start of co-culture. Once combined, the NK cells and A549-ACE2s were briefly spun down to bring the cells together and replaced in the incubator for 3 hours (killing assays) or 4 hours (NK activation assays) at 37°C. Following the incubation period, the cells were washed with PBS and stained with the eFluor 780 viability dye for 25 minutes. For NK cell activation assays, the cells were stained for 30 minutes with a panel of markers against surface markers expressed on NK cells. All assays were then fixed in 4% PFA (EIS, Cat. 15710) in PBS for 30 minutes, transferred to BSL2 facilities, washed, and stored overnight in 1% PFA in PBS at 4°C. The following day, activation assay samples were permeabilized (BD Biosciences, Cat. 340973), stained with a panel of intracellular markers, and collected on a Cytek Aurora for analysis. A table of flow cytometry reagents used can be found in Table S1.

#### Phenotypic analysis of SARS-CoV-2-infected and mock-infected cells

SARS-CoV-2-infected or mock-infected A549-ACE2 cells were harvested using TrypLE and transferred to a round-bottom 96-well plate. The cells were washed with PBS and stained with the eFluor 780 viability dye for 25 minutes. The cells were then stained for 30 minutes with a panel of markers against the ligands for 6 different receptors expressed by NK cells. They were then fixed in 4% PFA for 30 minutes, transferred to BSL2 facilities, washed, and stored overnight in 1% PFA in PBS at 4°C. To assay intracellular Nsp1 expression, cells were permeabilized and stained with an anti-Nsp1 primary antibody followed by a fluorescent secondary antibody (Table S1). Samples were collected on a Cytek Aurora for analysis.

#### Proteasomal and lysosomal degradation inhibition

Proteasome inhibitor MG-132 (abcam, Cat. ab141003) or lysosomal inhibitor BAF-A1 (Millipore Sigma, Cat. SML1661-.1ML) were added to mock- or SARS-CoV-2-infected cell cultures at 24 hours post-infection. As recommended by the manufacturers, MG-132 was used at a final concentration of 2 uM and BAF-A1 was used at a final concentration of 100 nM. NKG2D-L surface expression was measured by flow cytometry at 24 hours after inhibitor addition (48 hours post-infection).

#### RT-qPCR

RNA was extracted cells lysed in DNA/RNA Shield using RNA Clean & Concentrator kits (Zymo Research, Cat. R1018) and excess DNA was removed from the samples using the TURBO DNA-free Kit according to the manufacturer’s instructions (Fisher Scientific, Cat. AM1907). RT-qPCR reactions were prepared using the Invitrogen superscript III Platinum One Step qRT PCR Kit with ROX (Invitrogen, Cat. 11745500) and primer/probe Taqman assays ordered from Thermo Scientific (see key resources table). The QuantStudio 3 Real-Time PCR System was used to quantify transcript levels (Thermo Fisher, Cat. A28567). Three technical replicates of each sample were measured and all samples were normalized to an endogenous control (*18S*).

#### SARS-CoV-2 protein plasmids

Plasmids encoding individual SARS-CoV-2 proteins and GFP were obtained from the Qi lab at Stanford University. Each plasmid included Strep Tag II, allowing for identification of transfected cells that successfully expressed the protein of interest.

#### Transient transfection of A549-ACE2 and 293T

A549-ACE2s or 293Ts were plated the day before transfection in 24-well plates at a concentration of 75,000 cells per well. Plasmids were transfected into cells with the aid of FugeneHD (Promega, Cat. E2311) using a ratio of 4 uL FugeneHD per 1 ug of plasmid DNA. Four technical replicates of each transfection were performed. The cells were then placed into a 37°C incubator for 48 hours.

#### Transient transfection of K562

K562 cells were plated the day before transfection in 24-well plates at a concentration of 200,000 cells per well. The Nsp1 plasmid was transfected into the cells with the aid of Lipfoctamine 3000 (ThermoFisher Scientific, Cat. L3000001) per the manufacturer’s instructions. The cells were then placed into a 37°C incubator for 48 hours.

#### Ligand profiling of transfected cells

48 hours after transfection, transfected cells were harvested and transferred to a 96-well plate for flow cytometry staining. Cells were stained with the eFluor 780 viability dye and a panel of fluorescent antibodies against NKG2D-L, DNAM-1-L, CD54, and MHC class I (Table S1) before being fixed in 4% PFA for 15 minutes. Fixed cells were permeabilized and stained with a primary antibody against Strep Tag II, washed, and stained with a fluorescent secondary antibody against the primary antibody. Our primary antibody against Strep Tag II is a polyclonal rabbit IgG; therefore, our anti-rabbit secondary antibody was able to detect this primary antibody without detecting any of our surface antibodies, which are all mouse IgG. Cells were then analyzed on a CyTek Aurora.

#### Brefeldin A treatment of A549-ACE2s

A549-ACE2s were cultured in D10 alone or D10 supplemented with either 0.5x or 1x Brefeldin A (eBioscience, Cat. 00-4506-51) for 24 or 48 hours and expression of NK cell receptor ligands was expressed by spectral cytometry.

#### ELISA quantification of soluble MICA and soluble ULBP-2

Supernatants from mock or SARS-CoV-2 infected A549-ACE2s were harvested 48 hours post-infection. Triton-X 100 was added to the supernatants to a final concentration of 1% for inactivation of virus and samples were stored at −80°C until use. ELISAs were performed using the Human MICA DuoSet ELISA (R&D Systems, Cat. DY1800) and Human ULBP-2 DuoSet ELISA (R&D Systems, Cat. DY1298) kits according to the manufacturer’s instructions. 1% Triton-X 100 (Sigma-Aldrich, Cat. T9284-100ML) was added to the standards (prior to serial dilution) to account for effects of the inactivation reagent on soluble protein concentration.

#### Transfected cell killing assay

IL-2-activated NK cells were co-cultured for 3 hours at an E:T ratio of 5:1 with A549-ACE2s that had been transfected 48 hours earlier with either one of the SARS-CoV-2 proteins shown (Nsp1, Nsp2, Nsp6, Nsp7, Nsp9, Nsp10, Nsp13, Nsp15, ORF3a, Membrane, or ORF7a) or GFP. Following the incubation period, the cells were washed with PBS and stained with the eFluor 780 viability dye and an antibody against surface NKG2D-L before being fixed in 4% PFA for 15 minutes. Fixed cells were permeabilized and stained with a primary antibody against Strep Tag II, washed, and stained with a fluorescent secondary antibody against the primary antibody. Samples were then analyzed on a CyTek Aurora.

The proteins transfected in this experiment were selected randomly from a list of all SARS-CoV-2 plasmids for which NKG2D-L expression had been previously measured. Proteins that transfected with an efficiency of <2% were excluded from analysis. Killing assays using transfected SARS-CoV-2 proteins were performed in 4 batches using the same healthy donors, reagents, equipment, and cytometer. The batches were as follows: 1) Nsp9, Nsp13, ORF7a; 2) Nsp2, Nsp6, Nsp7, ORF3a; 3) Nsp10, Nsp15; 4) Nsp1, GFP (repeated 3 times).

### Quantification and statistical analysis

Flow cytometry data visualization was performed using FlowJo v10.7.1. Figures were generated in R using the *ggplot2* package. Colors for figures were generated using the *tayloRswift* package. Statistical analyses were performed as described in figure legends and plotted using the R *ggpubr* package.

## Data Availability

No new code was generated during this study. All data are available upon reasonable request directed to the lead contact. Any additional information required to reanalyze the data reported in this paper is available from the lead contact upon request.

## Supplemental information

Document S1. Figures S1–S8 and Table S1

Document S2. Article plus supplemental information

## References

[1] French A.R., Yokoyama W.M. (2003). Natural killer cells and viral infections. Curr. Opin. Immunol..

[2] Brandstadter J.D., Yang Y. (2011). Natural killer cell responses to viral infection. J. Innate Immun..

[3] Björkström N.K., Strunz B., Ljunggren H.-G. (2022). Natural killer cells in antiviral immunity. Nat. Rev. Immunol..

[4] Waggoner S.N., Reighard S.D., Gyurova I.E., Cranert S.A., Mahl S.E., Karmele E.P., McNally J.P., Moran M.T., Brooks T.R., Yaqoob F., Rydyznski C.E. (2016). Roles of natural killer cells in antiviral immunity. Curr. Opin. Virol..

[5] Giamarellos-Bourboulis E.J., Netea M.G., Rovina N., Akinosoglou K., Antoniadou A., Antonakos N., Damoraki G., Gkavogianni T., Adami M.-E., Katsaounou P. (2020). Complex immune dysregulation in COVID-19 patients with severe respiratory failure. Cell Host Microbe.

[6] Wilk A.J., Rustagi A., Zhao N.Q., Roque J., Martínez-Colón G.J., McKechnie J.L., Ivison G.T., Ranganath T., Vergara R., Hollis T. (2020). A single-cell atlas of the peripheral immune response in patients with severe COVID-19. Nat. Med..

[7] Wilk A.J., Lee M.J., Wei B., Parks B., Pi R., Martínez-Colón G.J., Ranganath T., Zhao N.Q., Taylor S., Becker W. (2021). Multi-omic profiling reveals widespread dysregulation of innate immunity and hematopoiesis in COVID-19. J. Exp. Med..

[8] Chen G., Wu D., Guo W., Cao Y., Huang D., Wang H., Wang T., Zhang X., Chen H., Yu H. (2020). Clinical and immunological features of severe and moderate coronavirus disease 2019. J. Clin. Invest..

[9] Maucourant C., Filipovic I., Ponzetta A., Aleman S., Cornillet M., Hertwig L., Strunz B., Lentini A., Reinius B., Brownlie D. (2020). Natural killer cell immunotypes related to COVID-19 disease severity. Sci. Immunol..

[10] Varchetta S., Mele D., Oliviero B., Mantovani S., Ludovisi S., Cerino A., Bruno R., Castelli A., Mosconi M., Vecchia M. (2021). Unique immunological profile in patients with COVID-19. Cell. Mol. Immunol..

[11] Zheng M., Gao Y., Wang G., Song G., Liu S., Sun D., Xu Y., Tian Z. (2020). Functional exhaustion of antiviral lymphocytes in COVID-19 patients. Cell. Mol. Immunol..

[12] Liu C., Martins A.J., Lau W.W., Rachmaninoff N., Chen J., Imberti L., Mostaghimi D., Fink D.L., Burbelo P.D., Dobbs K. (2021). Time-resolved systems immunology reveals a late juncture linked to fatal COVID-19. Cell.

[13] Osman M., Faridi R.M., Sligl W., Shabani-Rad M.-T., Dharmani-Khan P., Parker A., Kalra A., Tripathi M.B., Storek J., Cohen Tervaert J.W., Khan F.M. (2020). Impaired natural killer cell counts and cytolytic activity in patients with severe COVID-19. Blood Adv..

[14] Chua R.L., Lukassen S., Trump S., Hennig B.P., Wendisch D., Pott F., Debnath O., Thürmann L., Kurth F., Völker M.T. (2020). COVID-19 severity correlates with airway epithelium-immune cell interactions identified by single-cell analysis. Nat. Biotechnol..

[15] Leem G., Cheon S., Lee H., Choi S.J., Jeong S., Kim E.-S., Jeong H.W., Jeong H., Park S.-H., Kim Y.-S., Shin E.C. (2021). Abnormality in the NK-cell population is prolonged in severe COVID-19 patients. J. Allergy Clin. Immunol..

[16] Krämer B., Knoll R., Bonaguro L., ToVinh M., Raabe J., Astaburuaga-García R., Schulte-Schrepping J., Kaiser K.M., Rieke G.J., Bischoff J. (2021). Early IFN-α signatures and persistent dysfunction are distinguishing features of NK cells in severe COVID-19. Immunity.

[17] Bozzano F., Dentone C., Perrone C., Di Biagio A., Fenoglio D., Parodi A., Mikulska M., Bruzzone B., Giacobbe D.R., Vena A. (2021). Extensive activation, tissue trafficking, turnover and functional impairment of NK cells in COVID-19 patients at disease onset associates with subsequent disease severity. PLoS Pathog..

[18] Hammer Q., Dunst J., Christ W., Picarazzi F., Wendorff M., Momayyezi P., Huhn O., Netskar H.K., Maleki K.T., García M. (2022). SARS-CoV-2 Nsp13 encodes for an HLA-E-stabilizing peptide that abrogates inhibition of NKG2A-expressing NK cells. Cell Rep..

[19] Witkowski M., Tizian C., Ferreira-Gomes M., Niemeyer D., Jones T.C., Heinrich F., Frischbutter S., Angermair S., Hohnstein T., Mattiola I. (2021). Untimely TGFβ responses in COVID-19 limit antiviral functions of NK cells. Nature.

[20] Fielding C.A., Sabberwal P., Williamson J.C., Greenwood E.J.D., Crozier T.W.M., Zelek W., Seow J., Graham C., Huettner I., Edgeworth J.D. (2022). SARS-CoV-2 host-shutoff impacts innate NK cell functions, but antibody-dependent NK activity is strongly activated through non-spike antibodies. Elife.

[21] Shah A.H., Sowrirajan B., Davis Z.B., Ward J.P., Campbell E.M., Planelles V., Barker E. (2010). Degranulation of natural killer cells following interaction with HIV-1-infected cells is hindered by downmodulation of NTB-A by Vpu. Cell Host Microbe.

[22] Sutherland C.L., Chalupny N.J., Schooley K., VandenBos T., Kubin M., Cosman D. (2002). UL16-binding proteins, novel MHC class I-related proteins, bind to NKG2D and activate multiple signaling pathways in primary NK cells. J. Immunol..

[23] Wu J., Chalupny N.J., Manley T.J., Riddell S.R., Cosman D., Spies T. (2003). Intracellular retention of the MHC class I-related chain B ligand of NKG2D by the human cytomegalovirus UL16 glycoprotein. J. Immunol..

[24] Slavuljica I., Krmpotić A., Jonjić S. (2011). Manipulation of NKG2D ligands by cytomegaloviruses: impact on innate and adaptive immune response. Front. Immunol..

[25] Fittje P., Hœlzemer A., Garcia-Beltran W.F., Vollmers S., Niehrs A., Hagemann K., Martrus G., Körner C., Kirchhoff F., Sauter D., Altfeld M. (2022). HIV-1 Nef-mediated downregulation of CD155 results in viral restriction by KIR2DL5+ NK cells. PLoS Pathog..

[26] Ward J., Bonaparte M., Sacks J., Guterman J., Fogli M., Mavilio D., Barker E. (2007). HIV modulates the expression of ligands important in triggering natural killer cell cytotoxic responses on infected primary T-cell blasts. Blood.

[27] Zhao N.Q., Ferreira A.-M., Grant P.M., Holmes S., Blish C.A. (2020). Treated HIV infection alters phenotype but not HIV-specific function of peripheral blood natural killer cells. Front. Immunol..

[28] Fielding C.A., Weekes M.P., Nobre L.V., Ruckova E., Wilkie G.S., Paulo J.A., Chang C., Suárez N.M., Davies J.A., Antrobus R. (2017). Control of immune ligands by members of a cytomegalovirus gene expansion suppresses natural killer cell activation. Elife.

[29] Welte S.A., Sinzger C., Lutz S.Z., Singh-Jasuja H., Sampaio K.L., Eknigk U., Rammensee H.-G., Steinle A. (2003). Selective intracellular retention of virally induced NKG2D ligands by the human cytomegalovirus UL16 glycoprotein. Eur. J. Immunol..

[30] Richard J., Sindhu S., Pham T.N.Q., Belzile J.-P., Cohen E.A. (2010). HIV-1 Vpr up-regulates expression of ligands for the activating NKG2D receptor and promotes NK cell-mediated killing. Blood.

[31] Fielding C.A., Aicheler R., Stanton R.J., Wang E.C.Y., Han S., Seirafian S., Davies J., McSharry B.P., Weekes M.P., Antrobus P.R. (2014). Two novel human cytomegalovirus NK cell evasion functions target MICA for lysosomal degradation. PLoS Pathog..

[32] Fernández-Messina L., Reyburn H.T., Valés-Gómez M. (2016). A short half-life of ULBP1 at the cell surface due to internalization and proteosomal degradation. Immunol. Cell Biol..

[33] Klein S., Cortese M., Winter S.L., Wachsmuth-Melm M., Neufeldt C.J., Cerikan B., Stanifer M.L., Boulant S., Bartenschlager R., Chlanda P. (2020). SARS-CoV-2 structure and replication characterized by in situ cryo-electron tomography. Nat. Commun..

[34] Xie X., Muruato A., Lokugamage K.G., Narayanan K., Zhang X., Zou J., Liu J., Schindewolf C., Bopp N.E., Aguilar P.V. (2020). An infectious cDNA clone of SARS-CoV-2. Cell Host Microbe.

[35] Ogando N.S., Dalebout T.J., Zevenhoven-Dobbe J.C., Limpens R.W.A., van der Meer Y., Caly L. (2020). Bárcena, M.,et al. SARS-coronavirus-2 replication in Vero E6 cells: replication kinetics, rapid adaptation and cytopathology. J. Gen. Virol..

[36] Cheemarla, N.R., Watkins, T.A., Mihaylova, V.T., Wang, B., Zhao, D., and Wang, G. (2021). Dynamic innate immune response determines susceptibility to SARS-CoV-2 infection and early replication kinetics. J. Exp. Med. 218. 10.1084/jem.20210583 PMC8210587 34128960

[37] Arshad N., Laurent-Rolle M., Ahmed W.S., Hsu J.C.-C., Mitchell S.M., Pawlak J., Sengupta D., Biswas K.H., Cresswell P. (2022). SARS-CoV-2 accessory proteins ORF7a and ORF3a use distinct mechanisms to downregulate MHC-I surface expression. bioRxiv.

[38] Yoo J.-S., Sasaki M., Cho S.X., Kasuga Y., Zhu B., Ouda R., Orba Y., de Figueiredo P., Sawa H., Kobayashi K.S. (2021). SARS-CoV-2 inhibits induction of the MHC class I pathway by targeting the STAT1-IRF1-NLRC5 axis. Nat. Commun..

[39] Zhang Y., Chen Y., Li Y., Huang F., Luo B., Yuan Y., Xia B., Ma X., Yang T., Yu F. (2021). The ORF8 protein of SARS-CoV-2 mediates immune evasion through down-regulating MHC-Ι. Proc. Natl. Acad. Sci. USA.

[40] Mariano G., Farthing R.J., Lale-Farjat S.L.M., Bergeron J.R.C. (2020). Structural characterization of SARS-CoV-2: where we are, and where we need to Be. Front. Mol. Biosci..

[41] Naqvi A.A.T., Fatima K., Mohammad T., Fatima U., Singh I.K., Singh A., Atif S.M., Hariprasad G., Hasan G.M., Hassan M.I. (2020). Insights into SARS-CoV-2 genome, structure, evolution, pathogenesis and therapies: structural genomics approach. Biochim. Biophys. Acta, Mol. Basis Dis..

[42] Yadav R., Chaudhary J.K., Jain N., Chaudhary P.K., Khanra S., Dhamija P., Sharma A., Kumar A., Handu S. (2021). Role of structural and non-structural proteins and therapeutic targets of SARS-CoV-2 for COVID-19. Cells.

[43] Raj R. (2021). Analysis of non-structural proteins, NSPs of SARS-CoV-2 as targets for computational drug designing. Biochem. Biophys. Rep..

[44] Züst R., Cervantes-Barragán L., Kuri T., Blakqori G., Weber F., Ludewig B., Thiel V. (2007). Coronavirus non-structural protein 1 is a major pathogenicity factor: implications for the rational design of coronavirus vaccines. PLoS Pathog..

[45] Narayanan K., Huang C., Lokugamage K., Kamitani W., Ikegami T., Tseng C.-T.K., Makino S. (2008). Severe acute respiratory syndrome coronavirus nsp1 suppresses host gene expression, including that of type I interferon, in infected cells. J. Virol..

[46] Min Y.-Q., Mo Q., Wang J., Deng F., Wang H., Ning Y.-J. (2020). SARS-CoV-2 nsp1: bioinformatics, potential structural and functional features, and implications for drug/vaccine designs. Front. Microbiol..

[47] Vazquez C., Swanson S.E., Negatu S.G., Dittmar M., Miller J., Ramage H.R., Cherry S., Jurado K.A. (2021). SARS-CoV-2 viral proteins NSP1 and NSP13 inhibit interferon activation through distinct mechanisms. PLoS One.

[48] Kamitani W., Narayanan K., Huang C., Lokugamage K., Ikegami T., Ito N., Kubo H., Makino S. (2006). Severe acute respiratory syndrome coronavirus nsp1 protein suppresses host gene expression by promoting host mRNA degradation. Proc. Natl. Acad. Sci. USA.

[49] Schubert K., Karousis E.D., Jomaa A., Scaiola A., Echeverria B., Gurzeler L.-A., Leibundgut M., Thiel V., Mühlemann O., Ban N. (2020). SARS-CoV-2 Nsp1 binds the ribosomal mRNA channel to inhibit translation. Nat. Struct. Mol. Biol..

[50] Baugh R., Khalique H., Seymour L.W. (2020). Convergent evolution by cancer and viruses in evading the NKG2D immune response. Cancers.

[51] Raffaghello L., Prigione I., Airoldi I., Camoriano M., Levreri I., Gambini C., Pende D., Steinle A., Ferrone S., Pistoia V. (2004). Downregulation and/or release of NKG2D ligands as immune evasion strategy of human neuroblastoma. Neoplasia.

[52] Toledano T., Vitenshtein A., Stern-Ginossar N., Seidel E., Mandelboim O. (2018). Decay of the stress-induced ligand MICA is controlled by the expression of an alternative 3’ untranslated region. J. Immunol..

[53] Yarzabek B., Zaitouna A.J., Olson E., Silva G.N., Geng J., Geretz A., Thomas R., Krishnakumar S., Ramon D.S., Raghavan M. (2018). Variations in HLA-B cell surface expression, half-life and extracellular antigen receptivity. Elife.

[54] Braun M., Pietsch P., Zepp A., Schrör K., Baumann G., Felix S.B. (1997). Regulation of tumor necrosis factor alpha- and interleukin-1-beta-induced induced adhesion molecule expression in human vascular smooth muscle cells by cAMP. Arterioscler. Thromb. Vasc. Biol..

[55] Lanier L.L. (2015). NKG2D receptor and its ligands in host defense. Cancer Immunol. Res..

[56] Ward J., Davis Z., DeHart J., Zimmerman E., Bosque A., Brunetta E., Mavilio D., Planelles V., Barker E. (2009). HIV-1 Vpr triggers natural killer cell-mediated lysis of infected cells through activation of the ATR-mediated DNA damage response. PLoS Pathog..

[57] Zdrenghea M.T., Telcian A.G., Laza-Stanca V., Bellettato C.M., Edwards M.R., Nikonova A., Khaitov M.R., Azimi N., Groh V., Mallia P. (2012). RSV infection modulates IL-15 production and MICA levels in respiratory epithelial cells. Eur. Respir. J..

[58] Borchers M.T., Harris N.L., Wesselkamper S.C., Vitucci M., Cosman D. (2006). NKG2D ligands are expressed on stressed human airway epithelial cells. Am. J. Physiol. Lung Cell Mol. Physiol..

[59] Liao M., Liu Y., Yuan J., Wen Y., Xu G., Zhao J., Cheng L., Li J., Wang X., Wang F. (2020). Single-cell landscape of bronchoalveolar immune cells in patients with COVID-19. Nat. Med..

[60] Huang W., Li M., Luo G., Wu X., Su B., Zhao L., Zhang S., Chen X., Jia M., Zhu J. (2021). The inflammatory factors associated with disease severity to predict COVID-19 progression. J. Immunol..

[61] Brownlie D., Rødahl I., Varnaite R., Asgeirsson H., Glans H., Falck-Jones S., Vangeti S., Buggert M., Ljunggren H.-G., Michaëlsson J. (2022). Comparison of lung-homing receptor expression and activation profiles on NK cell and T cell subsets in COVID-19 and influenza. Front. Immunol..

[62] Yuan S., Balaji S., Lomakin I.B., Xiong Y. (2021). Coronavirus Nsp1: immune response suppression and protein expression inhibition. Front. Microbiol..

[63] Vora S.M., Fontana P., Mao T., Leger V., Zhang Y., Fu T.-M., Lieberman J., Gehrke L., Shi M., Wang L. (2022). Targeting stem-loop 1 of the SARS-CoV-2 5’ UTR to suppress viral translation and Nsp1 evasion. Proc. Natl. Acad. Sci. USA.

[64] Afsar M., Narayan R., Akhtar M.N., Das D., Rahil H., Nagaraj S.K., Eswarappa S.M., Tripathi S., Hussain T. (2022). Drug targeting Nsp1-ribosomal complex shows antiviral activity against SARS-CoV-2. Elife.

[65] Kärre K. (2008). Natural killer cell recognition of missing self. Nat. Immunol..

[66] Ljunggren H.G., Kärre K. (1990). In search of the “missing self”: MHC molecules and NK cell recognition. Immunol. Today.

[67] Vivier E., Raulet D.H., Moretta A., Caligiuri M.A., Zitvogel L., Lanier L.L., Yokoyama W.M., Ugolini S. (2011). Innate or adaptive immunity? The example of natural killer cells. Science.

[68] Barrow A.D., Colonna M. (2019). Exploiting NK cell surveillance pathways for cancer therapy. Cancers.

