# Supplementary material for: SARS-CoV-2 escapes direct NK cell killing through Nsp1-mediated downregulation of ligands for NKG2D
Source: Cell Rep. 2022 Dec 12;41(13):111892. doi: 10.1016/j.celrep.2022.111892 (PMC9742201; doi:10.1016/j.celrep.2022.111892)
Supplement: Document S1. Figures S1–S8 and Table S1 [file mmc1.pdf]

Cell Reports, Volume *41*

Supplemental information

SARS-CoV-2 escapes direct NK cell killing through Nsp1-mediated downregulation of ligands for NKG2D

Madeline J. Lee, Michelle W. Leong, Arjun Rustagi, Aimee Beck, Leiping Zeng, Susan Holmes, Lei S. Qi, and Catherine A. Blish

SUPPLEMENTAL DATA

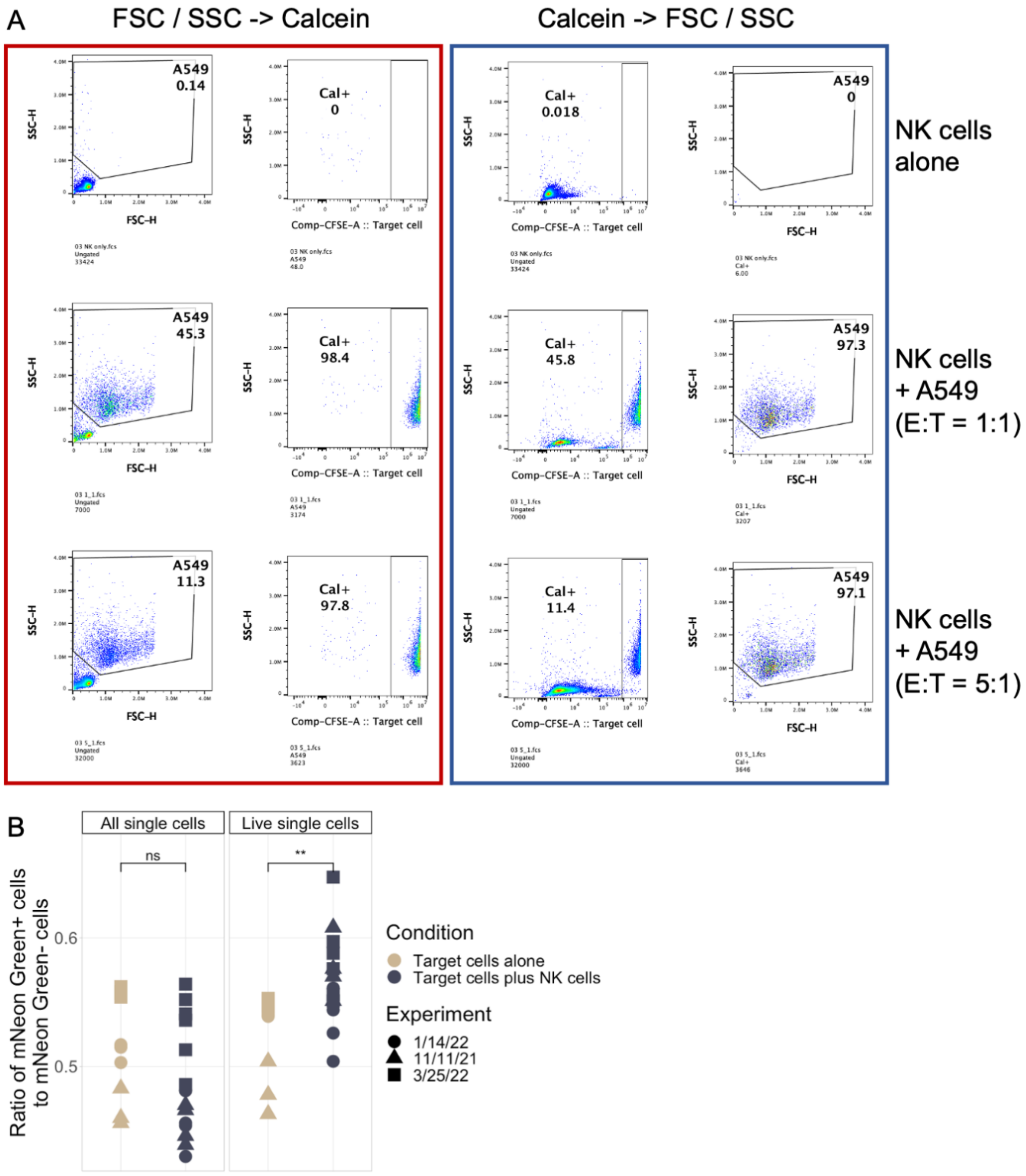

Figure S1: Identification of target cells in killing assays. Related to Figure 1. A) Representative flow plots showing that A549-ACE2s can be differentiated from NK cells by forward and side scatter alone. Target cells (A549-ACE2s) were labeled with Calcein, washed, and co-cultured with IL-2 pre-activated NK cells at various E:T ratios. Plots on the left side (red box) were gated first on FSC and SSC, then on Calcein expression; plots on the right side (blue box) were gated first on Calcein expression, then on FSC and SSC. B) Ratio of mNeon Green positive to mNeonGreen negative A549-ACE2s in the single cell compartment (left) and live single cell compartment (right).

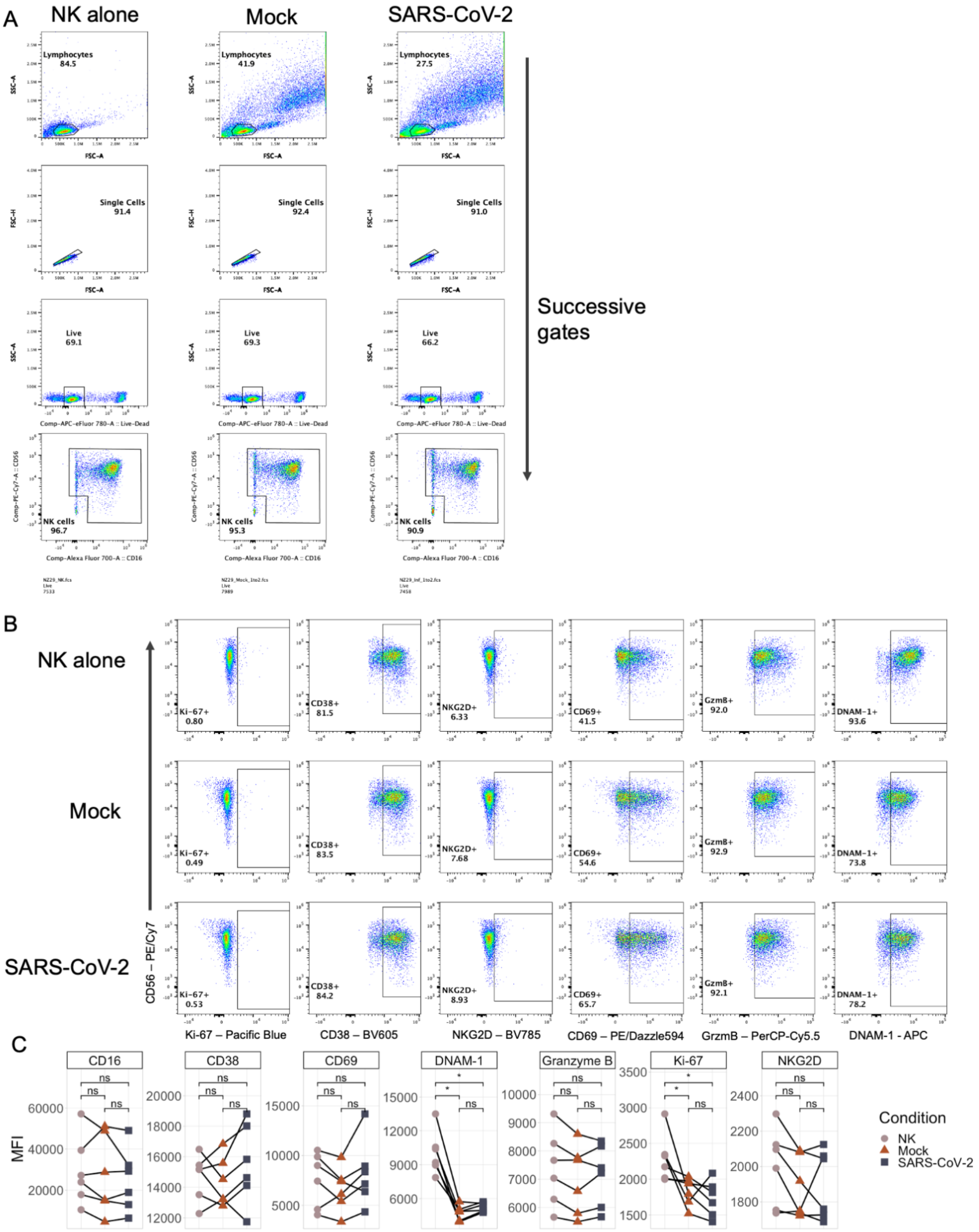

Figure S2: Effects of co-culture with mock or infected A549-ACE2 on NK cell phenotype. Related to Figure 1. A) Representative flow plots showing the gating scheme used to identify NK cells in functional assays with mock or SARS-CoV-2-infected A549-ACE2s. B) Representative flow plots showing the expression of various phenotypic and functional markers in NK cells across different co-culture conditions. C) Plots showing the mean fluorescence intensity (MFI) for seven different NK cell markers by flow cytometry upon culture with no targets, mock-infected targets, or SARS-CoV-2-infected targets.

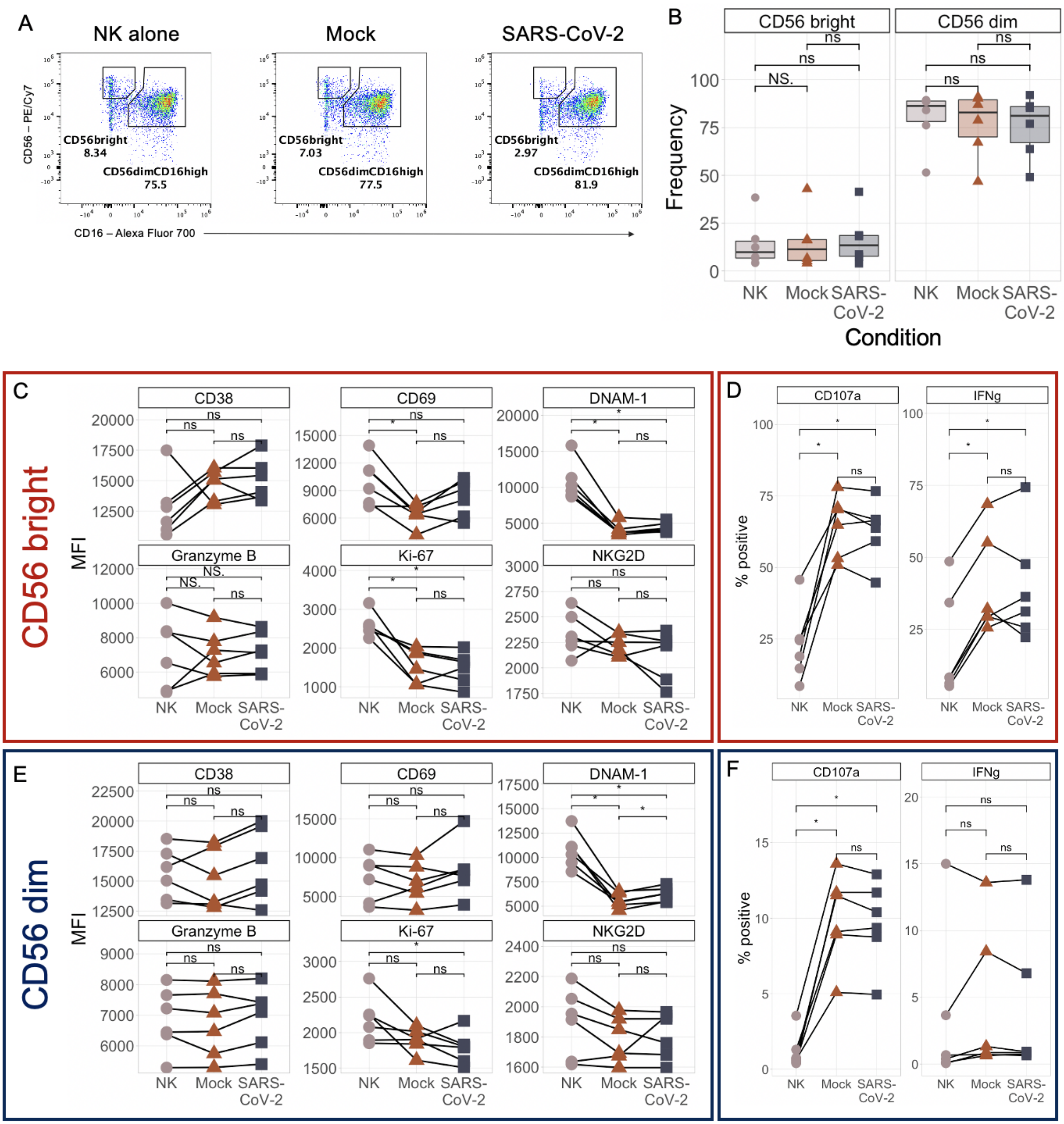

Figure S3: Effects of co-culture with mock or infected A549-ACE2 on NK cell subset frequencies and functions. Related to Figure 1. A-B) Representative flow plots (A) and boxplots (B) showing the frequency of CD56^{bright} and CD56^{dim} NK cells across different co-culture conditions. C) MFI of various phenotypic and functional markers on CD56^{bright} NK cells across infection conditions. D) Percentage of CD56^{bright} NK cells positive for CD107a (left) and IFNg (right) across infection conditions. E) MFI of various phenotypic and functional markers on CD56^{dim} NK cells across infection conditions. F) Percentage of CD56^{dim} NK cells positive for CD107a (left) and IFNg (right) across infection conditions. Lines in C-F represent individual donors. Significance values were calculated using a paired Wilcoxon signed-rank test with the Bonferroni correction for multiple hypothesis testing.

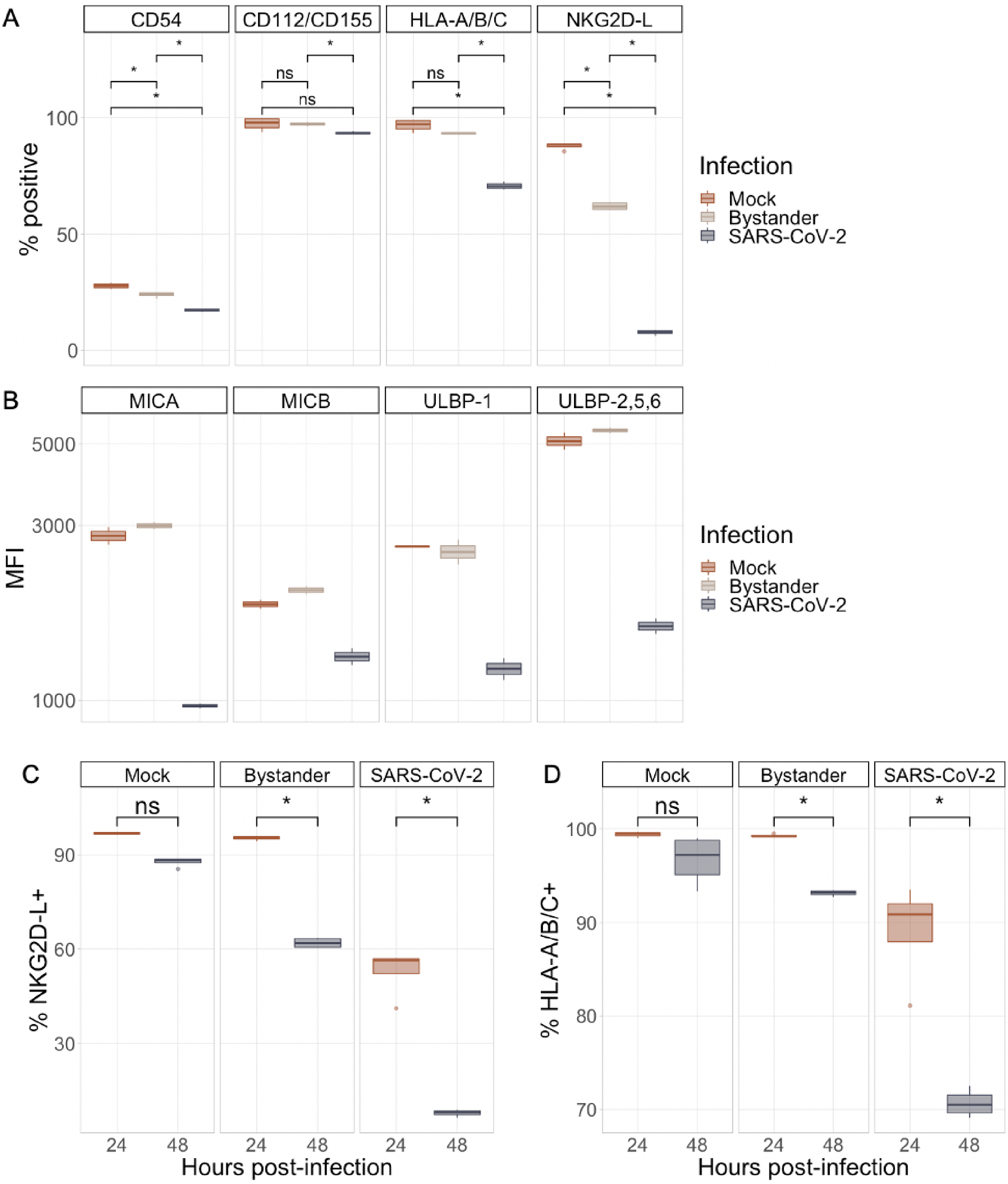

Figure S4: Expression of NK receptor ligands in SARS-CoV-2 infection. Related to Figure 2. A) Boxplots showing the percentage of A549-ACE2s expressing the ligands for various NK cell receptors across infection conditions at 48 hours post-infection. B) Mean fluorescence intensity (MFI) of the individual ligands comprising NKG2D-L across infection conditions at 48 hours post-infection. ULBPs 2, 5 and 6 are still grouped together as they are targeted by a single monoclonal antibody. C) Percentage of A549-ACE2s expressing NKG2D-L at 24 vs 48 hours post-infection across infection conditions. D) Percentage of A549-ACE2s expressing HLA-A/B/C at 24 vs 48 hours across infection conditions. All boxplots represent n=4 technical replicates.

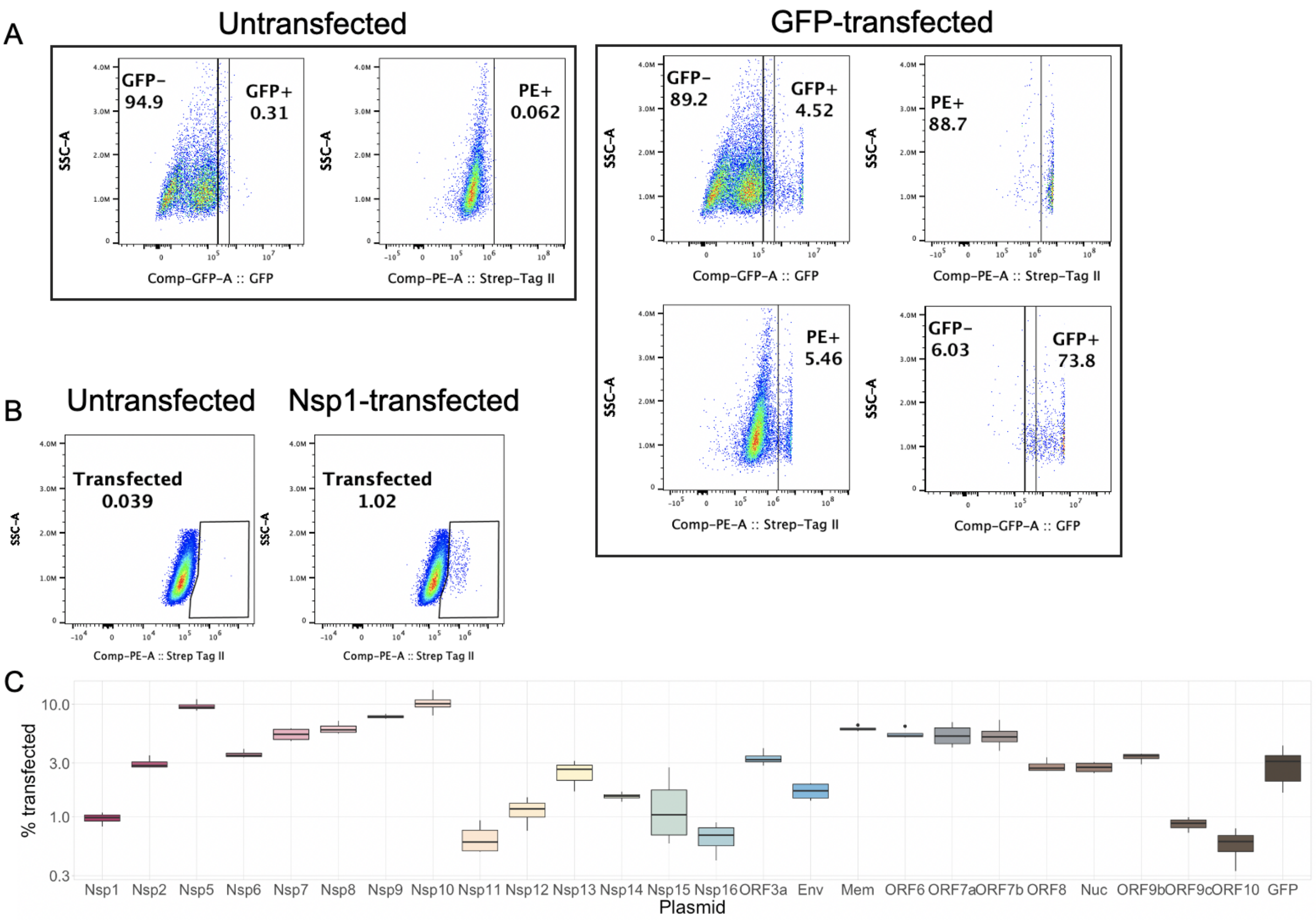

Figure S5: Validation of transfected cells identification. Related to Figure 4. A) Representative flow plots showing expression of GFP and Strep Tag II in mock-transfected versus GFP-transfected A549-ACE2s to illustrate overlap between GFP expression and Strep Tag II expression. B) Representative flow plots of Strep Tag II expression in mock-transfected vs Nsp1-transfected A549-ACE2s. C) Boxplots showing the percentage of cells positive for Strep Tag II by flow cytometry following transfection with plasmids encoding various proteins. All boxplots represent n=4 technical replicates for all plasmids.

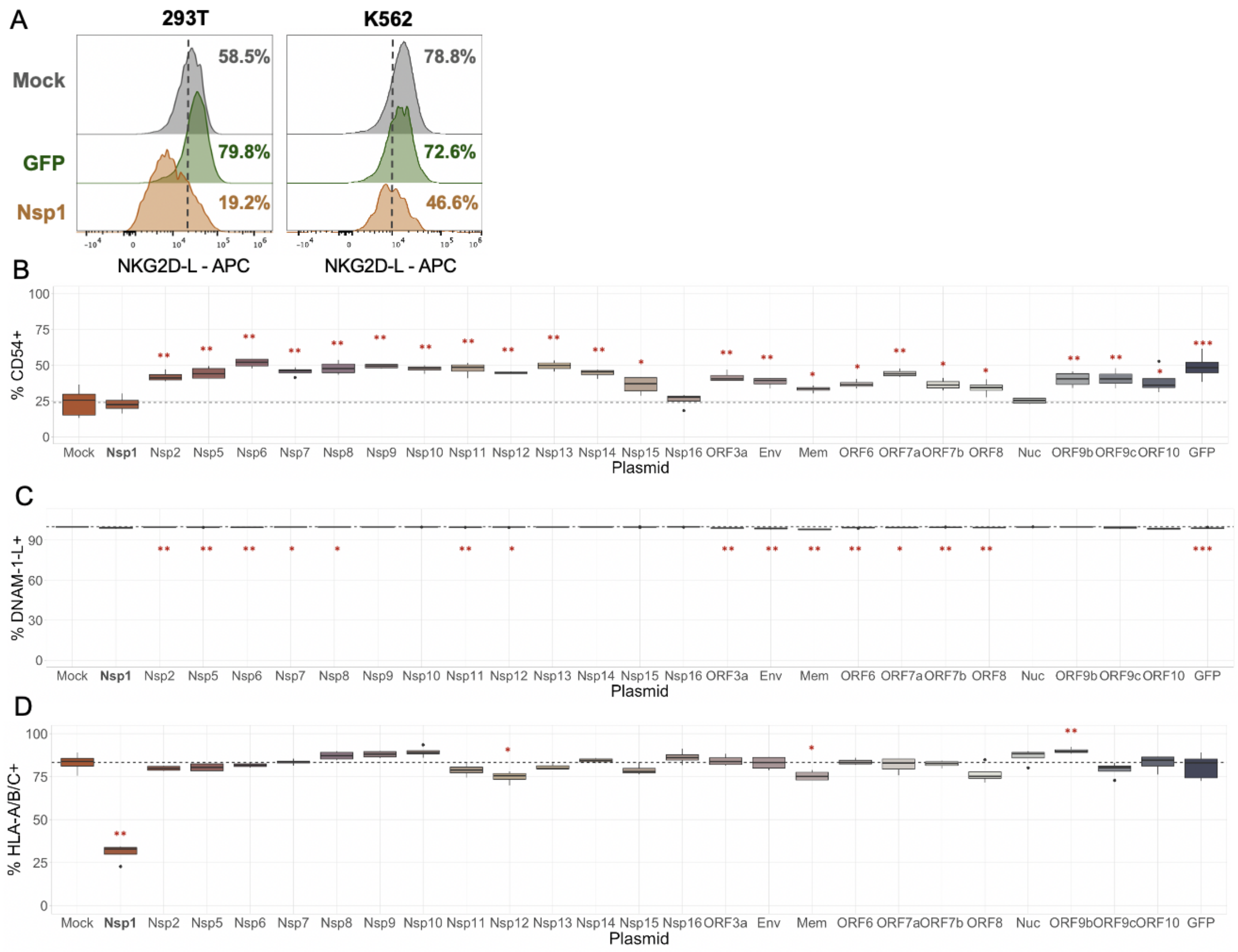

Figure S6: Frequency of ligand-expressing cells after transfection with SARS-CoV-2 proteins. Related to Figure 4. A) Histograms showing expression of NKG2D-L in 293T (left) or K562 (right) cells transfected with PBS (mock), GFP, or Nsp1. Dashed vertical line indicates threshold for positivity. Numbers to the right of the dashed line represent percentage of cells positive for NKG2D-L. B-D) Percentage of A549-ACE2s expressing CD54 (B), CD112/CD155 (C), or HLA-A/B/C by flow cytometry following transfection with plasmids encoding various proteins. Dashed lines represent the mean of mock-transfected samples expressing the ligand of interest. All boxplots represent n=4 technical replicates for all plasmids and n=8 for all "mock" samples. Asterisks represent significance values relative to mock-transfected samples.

Figure S7: Effects of Brefeldin-A treatment on A549-ACE2 NK receptor ligand expression

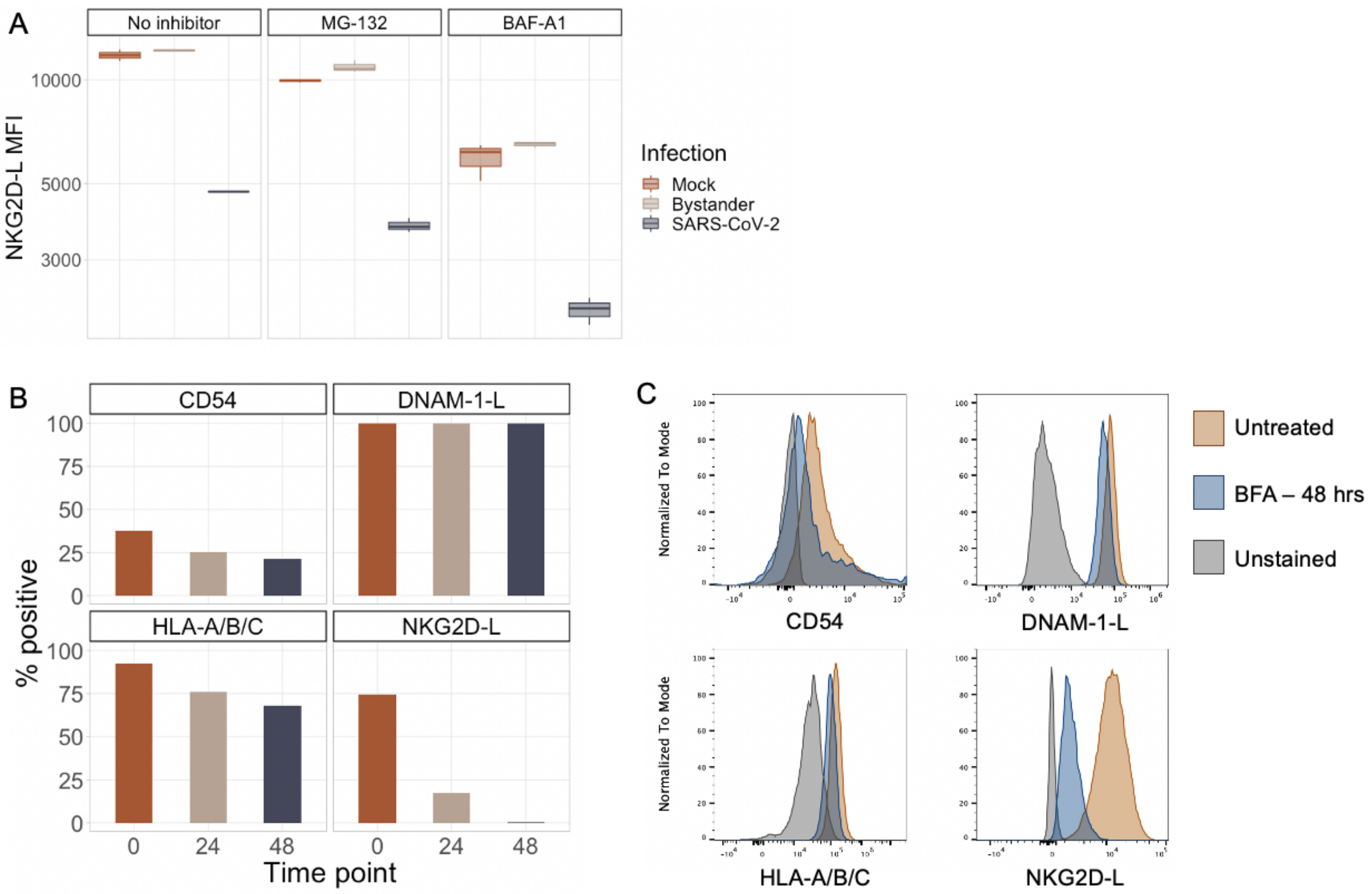

Figure S7: Effects of Brefeldin-A treatment on A549-ACE2 NK receptor ligand expression. A549-ACE2s were cultured for 24 or 48 hours with Brefeldin A or media alone. A) Percentage of A549-ACE2s expressing CD54, DNAM-1-L (CD112/CD155), HLA-A/B/C, or NKG2D-L after 24 or 48 hours with Brefeldin A or with no Brefeldin A (0 hr). Bars represent the mean of n=2 technical replicates. B) Representative histograms showing expression of these markers in BFA (1x)-treated or untreated A549-ACE2 after 48 hours.

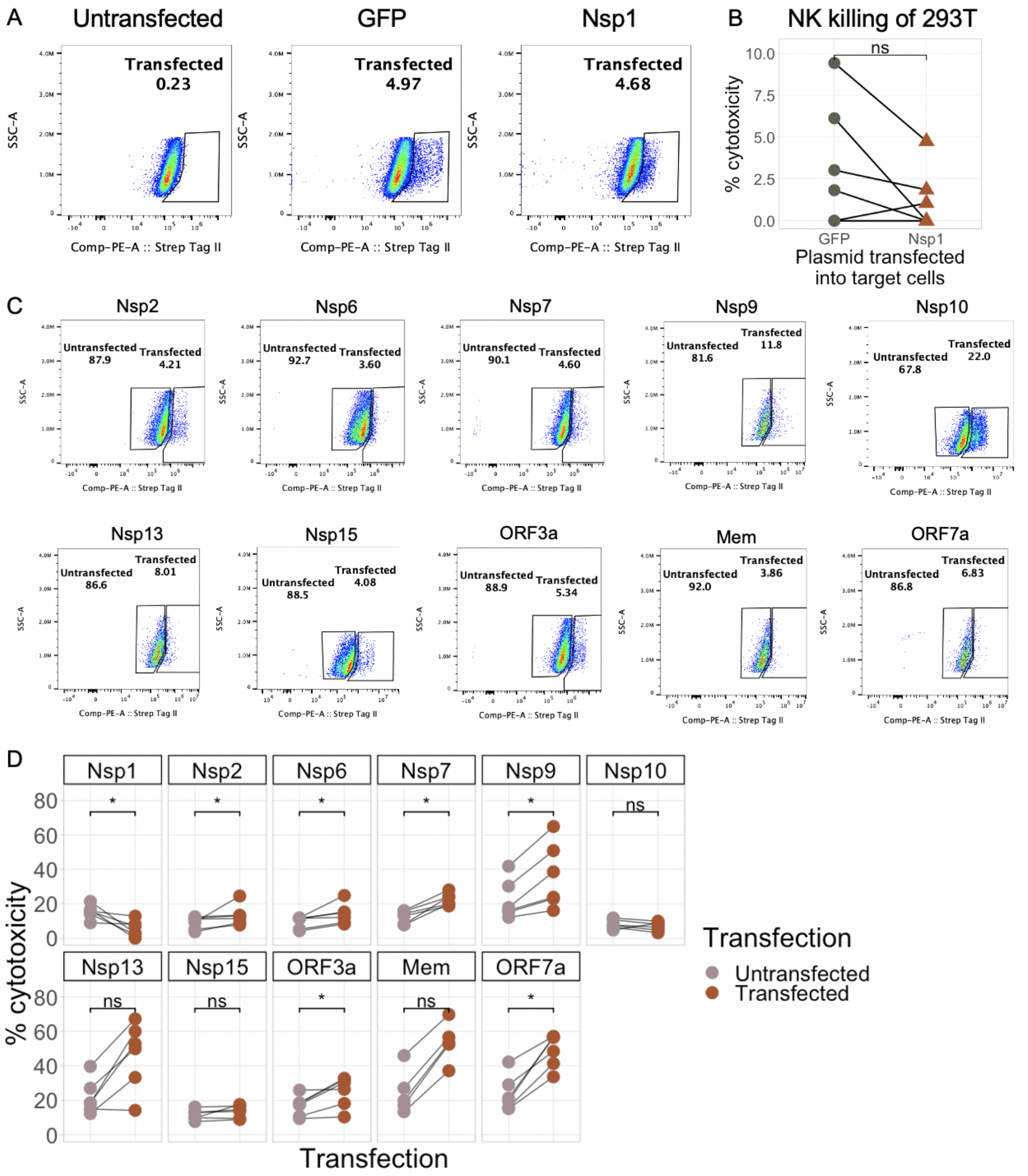

Figure S8: Identification of transfected cells in transfected cell killing assays. Related to Figure 7. A) Representative flow plots showing the percentage of transfected A549-ACE2s following transfection with Fugene only (left), GFP (middle), or Nsp1 (right). B) Representative flow plots showing the percentage of transfected A549-ACE2s following transfection with plasmids encoding the SARS-CoV-2 proteins shown in subfigure C. C) Background-subtracted target cell death among A549-ACE2s transfected with expression of various SARS-CoV-2 proteins and co-cultured with healthy NK cells (E:T = 5:1) for 3 hours. Lines in subfigure C represent individual donors. Significance values were determined using a paired Wilcoxon signed-rank test with the Bonferroni correction for multiple hypothesis testing.

Supplementary Table 1: Flow cytometry reagents

Antigen	Fluor	Clone	Host species	Manufacturer	RRID
CD56	PE-Cy7	HCD56	Mouse	Biolegend	RRID:AB_2149542
CD16	Alexa Fluor 700	3G8	Mouse	Biolegend	RRID:AB_493748
DNAM-1	APC	11A8	Mouse	Biolegend	RRID:AB_2561952
NKG2D	BV785	1D11	Mouse	Biolegend	RRID:AB_2728272
CD38	BV605	HIT2	Mouse	Biolegend	RRID:AB_2562915
CD69	PE-Dazzle594	FN50	Mouse	Biolegend	RRID:AB_2564276
CD107a	PE	H4A3	Mouse	Biolegend	RRID:AB_1186040
CD112	PerCP-Cy5.5	TX31	Mouse	Biolegend	RRID:AB_2565733
CD155	PerCP-Cy5.5	SKII.4	Mouse	Biolegend	RRID:AB_2565535
MICA	APC	159227	Mouse	R&D Systems	RRID:AB_2927482
MICB	APC	236511	Mouse	R&D Systems	RRID:AB_2297703
ULBP-1	APC	170818	Mouse	R&D Systems	RRID:AB_2923476
ULBP-2,5,6	APC	165903	Mouse	R&D Systems	RRID:AB_2257142
CD54	Alexa Fluor 700	HA58	Mouse	Biolegend	RRID:AB_2810560
HLA-A/B/C	Pacific Blue	W6/32	Mouse	Biolegend	RRID:AB_493668
Live/Dead	eFluor 780	N/A	N/A	ThermoFisher	NA
Annexin V	eFluor 450	N/A	N/A	Invitrogen	NA
7-AAD	7-AAD	N/A	N/A	Invitrogen	NA
Strep Tag II	N/A	Polyclonal	Rabbit	Abcam	RRID:AB_1524455
Nsp1	N/A	Polyclonal	Rabbit	Invitrogen	RRID:AB_2913171
Rabbit IgG	PE	Poly4064	Goat	Biolegend	RRID:AB_2563484

Table S1: Flow cytometry reagents. Relevant information regarding all antibodies and dyes used for flow cytometry experiments described in this manuscript.
